# Suspension culture improves iPSC expansion and pluripotency phenotype

**DOI:** 10.1186/s13287-023-03382-9

**Published:** 2023-06-06

**Authors:** Nerea Cuesta-Gomez, Kevin Verhoeff, Nidheesh Dadheech, Tiffany Dang, Ila Tewari Jasra, Mario Bermudez de Leon, Rena Pawlick, Braulio Marfil-Garza, Perveen Anwar, Haide Razavy, Patricio Adrián Zapata-Morin, Glen Jickling, Aducio Thiesen, Doug O’Gorman, Michael S. Kallos, A. M. James Shapiro

**Affiliations:** 1 Alberta Diabetes Institute, University of Alberta, Edmonton, AB T6G 2T9 Canada; 2 Department of Surgery, University of Alberta, Edmonton, AB T6G 2B7 Canada; 3 Pharmaceutical Production Research Facility (PPRF), Schulich School of Engineering, University of Calgary, Calgary, AB T2N1N4 Canada; 4 Department of Biomedical Engineering, Schulich School of Engineering, University of Calgary, Calgary, AB T2N1N4 Canada; 5 Department of Molecular Biology, Centro de Investigación Biomédica del Noreste, Instituto Mexicano del Seguro Social, 64720 Monterrey, Nuevo Leon Mexico; 6 National Institute of Medical Sciences and Nutrition Salvador Zubiran, 14080 Mexico City, Mexico; 7 CHRISTUS-LatAm Hub – Excellence and Innovation Center, 66260 Monterrey, Mexico; 8 Laboratory of Mycology and Phytopathology, Facultad de Ciencias Biológicas, Universidad Autónoma de Nuevo León, 66451 San Nicolás de los Garza, Nuevo León Mexico; 9 Department of Medicine, Division of Neurology, University of Alberta, Edmonton, AB T6G 2R3 Canada; 10 Department of Laboratory Medicine and Pathology, University of Alberta, Edmonton, AB T6G 2B7 Canada; 11 Clinical Islet Transplant Program, University of Alberta, Edmonton, AB T6G 2J3 Canada

**Keywords:** Human-induced pluripotent stem cells, iPSC, Stem cells, Cell therapy, Bioreactor, Expansion, Pluripotency

## Abstract

**Background:**

Induced pluripotent stem cells (iPSCs) offer potential to revolutionize regenerative medicine as a renewable source for islets, dopaminergic neurons, retinal cells, and cardiomyocytes. However, translation of these regenerative cell therapies requires cost-efficient mass manufacturing of high-quality human iPSCs. This study presents an improved three-dimensional Vertical-Wheel® bioreactor (3D suspension) cell expansion protocol with comparison to a two-dimensional (2D planar) protocol.

**Methods:**

Sendai virus transfection of human peripheral blood mononuclear cells was used to establish mycoplasma and virus free iPSC lines without common genetic duplications or deletions. iPSCs were then expanded under 2D planar and 3D suspension culture conditions. We comparatively evaluated cell expansion capacity, genetic integrity, pluripotency phenotype, and in vitro and in vivo pluripotency potential of iPSCs.

**Results:**

Expansion of iPSCs using Vertical-Wheel® bioreactors achieved 93.8-fold (IQR 30.2) growth compared to 19.1 (IQR 4.0) in 2D (*p* < 0.0022), the largest expansion potential reported to date over 5 days. 0.5 L Vertical-Wheel® bioreactors achieved similar expansion and further reduced iPSC production cost. 3D suspension expanded cells had increased proliferation, measured as Ki67^+^ expression using flow cytometry (3D: 69.4% [IQR 5.5%] vs. 2D: 57.4% [IQR 10.9%], *p* = 0.0022), and had a higher frequency of pluripotency marker (Oct4^+^Nanog^+^Sox2^+^) expression (3D: 94.3 [IQR 1.4] vs. 2D: 52.5% [IQR 5.6], *p* = 0.0079). q-PCR genetic analysis demonstrated a lack of duplications or deletions at the 8 most commonly mutated regions within iPSC lines after long-term passaging (> 25). 2D-cultured cells displayed a primed pluripotency phenotype, which transitioned to naïve after 3D-culture. Both 2D and 3D cells were capable of trilineage differentiation and following teratoma, 2D-expanded cells generated predominantly solid teratomas, while 3D-expanded cells produced more mature and predominantly cystic teratomas with lower Ki67^+^ expression within teratomas (3D: 16.7% [IQR 3.2%] vs.. 2D: 45.3% [IQR 3.0%], *p* = 0.002) in keeping with a naïve phenotype.

**Conclusion:**

This study demonstrates nearly 100-fold iPSC expansion over 5-days using our 3D suspension culture protocol in Vertical-Wheel® bioreactors, the largest cell growth reported to date. 3D expanded cells showed enhanced in vitro and in vivo pluripotency phenotype that may support more efficient scale-up strategies and safer clinical implementation.

**Supplementary Information:**

The online version contains supplementary material available at 10.1186/s13287-023-03382-9.

## Background

Human-induced pluripotent stem cells (iPSCs) possess the potential to revolutionize the field of regenerative medicine, offering the capacity to generate autologous tissues such as islets, cardiomyocytes, retinal cells, or dopaminergic neurons [1, 2, 3, 4, 5, 6, 7, 8, 9, 10, 11]. However, to implement iPSCs and their ensuing islet, cardiomyocyte, or other differentiated cell products clinically, up to 10^8^–10^10^ cells per patient would be required [12, 13]. Producing the required cells in a cost-effective and scalable manner remains a challenge. Further, to ensure cell product safety, expanded cells should ideally display a naïve pluripotency phenotype and maintain consistent differentiation capacity over time [12, 13, 14]. While substantial work has evaluated the ideal approach for iPSC generation [15, 16] and tissue differentiation [1, 2, 3, 4, 5, 6, 7, 8, 9, 10, 11], few studies have comparatively assessed iPSC expansion protocols in terms of scalability, pluripotency phenotype, and differentiation potential [17, 18, 19, 20, 21].

After the discovery of human iPSCs in 2007 [22], initial iPSC expansion and differentiation experiments utilized two-dimensional (2D) planar expansion using feeder layers or supporting extracellular matrices, which resulted in approximately tenfold expansion [1, 2, 6, 17, 23, 24, 25]. More recently, iPSC growth within three-dimensional (3D) suspension conditions using stirred suspension bioreactors has been suggested to provide a superior environment for expansion. This is due to improved mixing effects, which effectively distributes dissolved gasses and nutrients throughout the culture environment [18, 19, 20, 21]. While initial studies achieved 20- to 40-fold expansion using suspension culture, some authors cautioned that the introduction of complex hydrodynamic forces in the bioreactor could adversely affect cell viability and pluripotency [20, 26, 27, 28]. To mitigate these potentially deleterious effects, Vertical-Wheel® bioreactors have recently been investigated because of their unique geometry which reduces shear stress effects and improves vessel content homogenization [27, 28, 29, 30]. Despite the proposed benefits of suspension culture using Vertical-Wheel® bioreactors, protocol optimization with direct thorough comparison to 2D planar expansion techniques is needed. Additionally, while cells grown in both conditions are capable of differentiating into tissues of interest [1, 2, 3, 4, 5, 6, 7, 8, 9, 10, 11], comparison of their pluripotency phenotype remains elusive. Optimization of 3D suspension expansion protocols to achieve maximal iPSC expansion of homogeneous high-quality cells is essential to achieve adequate cell yield for safer, efficient, and cost-effective in-human clinical implementation of iPSC-derived cell therapies.

Herein, we present a modified iPSC expansion protocol using 3D suspension culture within Vertical-Wheel® bioreactors that achieves the largest cell expansion to date, in a single passage, while maintaining a high-quality cell product. We evaluate the expansion potential of this 3D suspension protocol in terms of growth kinetics, viability, genetic stability, pluripotency phenotype, in vitro and in vivo pluripotency against iPSCs expanded in 2D planar conditions. Results from this study elucidate opportunities and impediments for scalability of iPSC expansion to improve future clinical implementation of iPSC-derived cell therapies.

## Methods

### Experimental model and subject details

All procedures and protocols were approved by the Stem Cell Oversight Committee (SCOC), Canada, and the University of Alberta Institutional Health Research Ethics Board (PRO00084032). All animal protocols were conducted in accordance with the Canadian Council on Animal Care Guidelines and Policies with approval from the Animal Care and Use Committee (Health Sciences) for the University of Alberta. Manuscript reporting adheres to the ARRIVE guidelines for the reporting of animal experiments. Animals were euthanized under anesthesia (5% isoflurane) by a combination of thoracotomy and exsanguination. Patients recruited as blood sample donors provided written consent for the use of tissue, cell reprogramming, and result disclosure. All experiments were planned a priori and completed in technical and biological triplicate based on standard experimental procedures without exclusion of experimental groups. The scientist performing analysis was blinded to the group allocation of samples. Other confounders were not controlled for.

### Cell culture

Cell culture was completed using good manufacturing practice (GMP) compliant materials, where available, to replicate clinical conditions [31]. Cell processing was performed in a Class-II biocontainment compliant lab with the manipulation of cells taking place in a sterile environment with high efficiency particulate air filtration. Cells were maintained at 37 °C with 5% CO_2_ within humidified incubators.

#### Generation of induced pluripotent stem cell lines

In this study, 4 human iPSC lines were generated from peripheral blood mononuclear cells (PBMCs) from healthy donors (patient demographics in Additional file 1: Table S1). Donor blood (20.0 mL) was collected into BD vacutainer spray coated K2EDTA tubes (Thermo Fisher Scientific cat.13-680-61). Collected blood was diluted equally with Ca^2+^/Mg^2+^-free phosphate buffer solution (PBS, pH 7.2) with 2 mM ethylenediaminetetraacetic acid (EDTA; EMD Millipore cat. 324506). The PBS-Blood solution (20.0 mL) was carefully layered over top of 15.0 mL histopaque density gradient solution (Sigma, cat. 10771) in 2 tubes and centrifuged at 800 g for 30 min without breaks. The solution was washed with 20 mL PBS-EDTA and centrifuged at 300 g for 10 min to create a density gradient from which the PBMCs were isolated using a serological pipette. Isolated PBMCs were cultured in StemPro-34 Serum Free Complete Media (Gibco, cat. A14509) supplemented with human recombinant cytokines (10 ng/mL IL3, IL6, SCF and FLT3; R&D, cat. 203-GMP, 206-IL, 7466-SC, and 308E-GMP respectively) for 4 days. PBMCs were reprogrammed using the CytoTune iPS 2.0 Sendai Reprogramming Kit (Thermo Fisher Scientific cat. A16517), whereby 500,000 PBMCs were infected with the appropriate combination of Sendai virus particles (KOS, C-Myc, Klf4) for 24-h (h) as per manufacturer recommendations. This was followed by cell culture with StemPro-34 Complete Media, supplemented with human recombinant cytokines (10 ng/mL IL3, IL6, SCF and FLT3) for 2 days. Single floating virus infected cells were pooled and transitioned to BioLite cell culture treated plates (Thermo Fisher Scientific cat. 130181) coated with human recombinant vitronectin (rhVTN) as per manufacturer recommendations (Thermo Fisher Scientific cat. A27940) and grown with StemPro-34 Complete Media from days 3–6 with daily media replacement. From day 7 onwards, attached cells were cultured using StemFlex media (Thermo Fisher Scientific cat. A3349401). Between days 15–20, individual colonies (hereafter referred to as clones) were handpicked under 10 × phase objective (using ECHO inverted Rebel microscope and ECHO image acquisition application). Each clonal cell line was scrutinized for viral clearance and pluripotent stem cell quality control criteria (immunohistochemistry and flow cytometry for Oct4, Sox2, SSEA4, Nanog, Tra-1-81, and Tra-1-60, expression of alkaline phosphatase (ALP), and lack of duplications or deletions at the 8 most commonly mutated regions within iPSC lines) with the best clone used to establish an iPSC cell line for this study. During this process of colony development and expansion, the entire cell culture dish was imaged to assess colony position and number of colonies reprogramed using the Cell Observation System Biostudio-T microscope (Nikon, MLA10000); image acquisition and processing was performed using NIS-element AR version 5.30.02 (Nikon, MQS31000) combined with PCR-AR-02 iPSC Colony Area Package (Nikon, MQS60002) software.

#### Induced pluripotent stem cell culture maintenance

iPSC lines were maintained in 60 mm rhVTN coated tissue culture plates (Thermo Fisher Scientific cat. 130181) with StemFlex media. rhVTN plates in this study were used only once to maintain GMP compliance, but we have also been successful using this technique with reuse of plates for up to five passages. Cultures were monitored daily using a Nikon TE300 Inverted Fluorescence Phase Contrast Microscope. Upon 80% confluency, cells were subcultured. For subculture from rhVTN plates, StemFlex media was removed and plates were washed with 2.0 mL of PBS. PBS was removed, and plates were incubated for 2 min at 37 °C and 5% CO_2_ with CTS EDTA Versene Solution (Thermo Fisher Scientific, cat. A4239101) supplemented with 10 μM Rho-kinase inhibitor (RockI; Y-27632 STEMCell Technologies cat. 72304). After incubation, the EDTA solution was removed and detachment of the cells was performed with mechanical disruption using StemFlex media supplemented with 10 μM RockI. Cells were then collected into a 1.5 mL tube and spun down at 450 g for 2 min. The supernatant was removed, and cells were resuspended in culture media for subculture or used for experimental purposes.

Throughout this text, a cell passage is described as cell detachment from a culture dish in 2D planar conditions as described above, or dissociating clusters into single cells in 3D suspension conditions as described below. Quality control of the cell lines was routinely performed every 5 passages and prior to experimentation. Following each passage, cells were counted and viability was assessed using the Thermo Fisher Scientific Invitrogen Countess II AMQAX1000 Cell Counter. To accomplish this, 20 μL of single cell solution was combined with 20 μL of 0.4% trypan blue (Thermo Fisher Scientific cat. 15250061) and placed in a Countess cell counting chamber. Live cell numbers were used to calculate cell requirements for all processes.

#### Induced pluripotent stem cell expansion in 2D planar and 3D suspension conditions

Following cell passaging, iPSCs allocated for expansion were randomly assigned to 2D planar or 3D suspension conditions.

For 2D planar expansion 2 × 10^6^ live cells were seeded into 150 mm plates coated with geltrex (Thermo Fisher Scientific cat. A1413302) in 20 mL of StemFlex media with 10 μM RockI. Geltrex coating was prepared at 6 µg/mL concentration using cold DMEM-F12 media (Thermo Fisher Scientific cat. 10313021) and incubated for 1 h at 37 °C prior to cell seeding as per manufacturer recommendations. 24 h post-seeding, media was removed and 20.0 mL of fresh pre-warmed StemFlex without RockI was added. Media was replaced daily for 5 days during cell expansion. Media was collected daily to assess pH, glucose, lactate, lactate dehydrogenase, ammonia, and glutamine using the Cedex bio analyzer (Roche cat. 06395554001). Cells were lifted for evaluation by incubating them with CTS EDTA Versene Solution for 8 min at 37 °C.

The 3D suspension expansion protocol was modified from previously published reports by Borys et al. [27], Rohani et al. [32], and Dang et al. [28]. For expansion, 2 × 10^6^ live iPSCs were seeded into 0.1 L Vertical-Wheel® bioreactors (PBS Biotech Inc.) in 55.0 mL of pre-incubated StemFlex media with 10 μM RockI (day 0) with constant rotational speed of 60 revolution per minute (rpm). Pre-incubation of StemFlex media is critical to allow temperature and pH stabilization. After 24 h, clustered iPSCs were then supplemented with 45.0 mL of StemFlex media without RockI (day 1). On day 3, clusters were allowed to gravity settle and the upper 50.0 mL of StemFlex was replaced with 50.0 mL of fresh pre-incubated media. On day 5, clusters were harvested for experimental purposes or were dissociated for further expansion. Media was collected on days 2–5 for 3D suspension when cells would settle by gravity to allow media sampling.

For passaging of 3D suspension iPSCs, clusters were allowed to gravity settle and StemFlex was removed. Clusters were washed with 30 mL of PBS with 10 μM RockI and were allowed to gravity settle. Supernatant was removed and clusters were incubated within the bioreactor with 10.0 mL of StemPro Accutase enzyme supplemented with 10 μM RockI for 10 min at 20 rpm and 37 °C. Following incubation, clusters were immediately disrupted with mechanical forces aspirating the clusters up and down with a 10 mL serological pipette. Single cells were transferred into a 15-mL conical tube and centrifuged at 450 g for 2 min. Supernatant was discarded and cells were resuspended in 10 mL of StemFlex with 10 μM RockI. Finally, cells were counted for live and dead cells with trypan blue solution described above and subsequently cultured or prepared for experimental purposes. During media replenishment and exchanges, spent media was collected for assessment.

To further compare the efficiency of our expansion protocol to other 3D protocols, we replicated the protocol described by Dang et al. [28] that previously reported the highest fold expansion using Vertical-Wheel® bioreactors. To reduce inter-protocol variability, we cultured cells using StemFlex rather than the modified B8 media initially reported by Dang et al. [28]. In this protocol, 2 × 10^6^ live iPSCs were seeded into 0.1 L Vertical-Wheel® bioreactors (PBS Biotech Inc.) in 100.0 mL of StemFlex media with 10 μM RockI (day 0) with a constant rotational speed of 60 rpm (day 0). On days 3, 5 and 6, clusters were allowed to gravity settle and the upper 50 mL of StemFlex was replaced with 50 mL of fresh pre-warmed StemFlex. Cell counts and aggregate sizing samples were taken daily from the bioreactors to assess growth kinetics and aggregate morphology. On day 7, clusters were harvested for experimental purposes or were dissociated for further expansion.

#### Embryoid body formation

Embryoid body (EB) formation was performed using AggreWell 400 plates (STEMCell Technologies, cat. 34425) as per manufacturer instructions. Briefly, each well of the AggreWell 400 plates was rinsed twice with 2.0 mL of Anti-Adherence Rinsing Solution (STEMCell Technologies cat. 07010) followed by centrifugation at 1300 g for 5 min in a swinging bucket rotor fitted with plate holders. Anti-Adherence Rinsing Solution was replaced with 5.0 mL of DMEM/F12 before use. Following preparation of the AggreWell 400 plates, 2D planar cells or 3D suspension cells were lifted and/or dissociated and a single cell suspension in StemFlex media with 10 μM RockI was prepared. Next, 4 × 10^6^ cells were seeded per well and plates were incubated at 37 °C for 24 h. After 24 h, the EBs were gently collected from the AggreWell 400 plates by pipetting the media up and down with a wide bore 10 mL pipette and were transferred into a 50 mL conical tube where they were allowed to gravity settle. Supernatant containing StemFlex media with RockI was removed and EBs were resuspended into fresh StemFlex media and transferred into a 100 mm low adhesion plate. EBs were kept in culture for an additional 4 days with media change on day 3.

#### Trilineage differentiation

To evaluate the pluripotency potential of 2D planar and 3D suspension expanded cells, we completed trilineage differentiation using the Human Pluripotent Stem Cell Functional Identification Kit (R&D cat. SC027B). 2D planar cells were seeded into a geltrex-coated plate (or coverslip for immunohistochemistry samples) then grown and differentiated according to manufacturer instructions. We attempted to differentiate 3D suspension cell clusters by placing 2000 clusters into a 6-well suspension culture plate alongside 2.0 mL of differentiation media; unfortunately, the trilineage differentiation kits were not capable of maintaining cells in 3D culture leading to 100% cell death. Instead, 3D cell clusters were dissociated and seeded into a 60 mm rhVTN coated plate (or coverslip for immunohistochemistry samples), allowed to grow until appropriate confluency, and differentiated as per kit instructions (R&D cat. SC027B).

### Molecular biology 

#### Ribonucleic acid (RNA) extraction and reverse transcription

Prior to RNA extraction, all materials were cleaned with RNase AWAY to decontaminate surfaces (Thermo Fisher Scientific cat. 10328011). A pellet of a maximum of 5 × 10^6^ cells was lysed with 350 µL RLT buffer (Qiagen cat. 79216) and frozen at −80 °C until RNA extraction. Suspension of lysed cells in RLT buffer was thawed and cells were disrupted and homogenized using the QIAshredder system (Qiagen), and total RNA was then extracted with the RNeasy Mini Kit (Qiagen cat. 74104) according to the manufacturer instructions. Concentration and purity of the isolated RNA samples were evaluated using spectrophotometry with the Multiskan SkyHigh Microplate Spectrophotometer and µdrop plate (Thermo Fisher Scientific cat. A51119600DPC) by assessing the 260/280 nm and 260/230 nm absorption of samples. Samples were then stored at −80 °C until needed; RNA was quantified after each defrost.

RNA was reverse-transcribed using the RevertAid First Strand cDNA Synthesis Kit as per manufacturer guidelines (Thermo Fisher Scientific, cat. K1621). Complement DNA (cDNA) was stored at −20 °C until required for PCR.

#### Polymerase chain reaction (PCR)

cDNAs were thawed and combined with PCR mix as described in Additional file 1: Table S2. GoTaq G2 Hot Start Colorless Master Mix (Promega, cat. M7422) was used alongside forward and reverse primers as described in Additional file 1: Table S3. Samples were placed in a thermocycler and underwent the sequence specified in Additional file 1: Table S4. For mycoplasma detection, Mycoplasma PCR Detection Kit (ABM cat. G238) was used as per manufacturer instructions. Samples were loaded into a 2% agarose gel (Invitrogen cat. 16520-050) with GelRed 6X loading Dye (RCD cat. 41003) and ran for 35 min at 100 V. Gels were visualized using the Image Quant 300 Gel documentation station under ultraviolet light.

#### Genomic DNA extraction

Whole genomic DNA (gDNA) was extracted by lysing a maximum of 5 × 10^6^ cells for 18–24 h at 55 °C in 487.5 µL TENS buffer (10 mM Tris–HCl (Sigma cat. T3253) pH 8.0, 25 mM EDTA (Sigma cat. 324506) pH 7.5, 100 mM NaCl (Sigma cat. S1679), 0.5% SDS (Sigma cat. 71736)) with 12.5 µL of proteinase K (20 mg/mL; Sigma cat. 70663-4). Proteins were precipitated using 250 µL of 6 M NaCl followed by centrifugation for 5 min at 12,000 g. The supernatant was recovered, and gDNA was precipitated with 900 µL isopropanol followed by centrifugation at 12,000 g for 10 min. The pellet was collected and washed with cold 70% EtOH and allowed to dry. gDNA was resuspended in 50 µL of TE buffer (10 mM Tris–HCl pH 8.0, 1 mM EDTA pH 8.0) and purity was assessed using the Multiskan SkyHigh Microplate Spectrophotometer and µdrop plate (Thermo Fisher Scientific). Sample purity was measured by determining the 260/280 nm and 260/230 nm absorption ratios with samples achieving 1.7–2.0 and 2.0–2.2, respectively, being used.

#### Quantitative PCR (q-PCR)

For genetic analysis, reactions were set up in 96-well plates using the hPSC Genetic Analysis Kit (STEMCell technologies cat. 07550) as per manufacturer instructions. q-PCR was performed using the StepOnePlus Real-Time PCR System (Thermo Fisher Scientific cat. 4376600) and gDNA was amplified as per Additional file 1: Table S5. Samples were analyzed using chromosome 4p as reference using the calculations below:

$$\Delta {C}_{t}={C}_{t} \left(\mathrm{target\,region}\right)- {C}_{t}\left(\mathrm{chromosome} 4p\right)$$

ΔΔC_t_ was calculated by subtracting the average ΔC_t_ of the gDNA from the control sample (supplied with the kit) for each sample to be tested.

$$\Delta {\Delta C}_{t}={C}_{t} \left(\Delta \mathrm{sample\,of\,interest}\right)- {\Delta C}_{t}\left(\mathrm{chromosome} 4p\right)$$

Data were represented as 2^(−ΔΔCT)^ × 2, which enables the visualization of copy number for the specific chromosomal regions of each sample.

$$\mathrm{Copy\,number}= {2}^{(- \Delta \Delta {C}_{t})}\cdot 2$$

The median of the RQ values of each sample was used for statistical analysis.

#### Quantitative reverse transcription PCR (qRT-PCR)

Custom designed gene TaqMan Low Density Array Cards were used as per manufacturer instructions (Thermo Fisher Scientific cat. 4342253); gene array set up is described in Additional file 1: Table S6. Briefly, 500 ng of cDNA was combined with 55 μL of nuclease free water and 55 μL TaqMan Universal PCR Master Mix (Thermo Fisher Scientific cat. 4305719). The combined solution was loaded into the gene array cards, centrifuged, and processed using the FAST-384 well array program via the QuantStudio 12 K Flex Real-Time PCR system.

Alternatively, pairs of primers were designed (sequences detailed in Additional file 1: Table S7) to quantify the amount of specific cDNA by SYBR Green qRT-PCR (Thermo Fisher Scientific cat. 4385612). qRT-PCR assay was performed using the Applied Biosystems 7900HC Fast Real-Time PCR Systems detection system (Applied Biosystems). Samples were analyzed using B2M as reference for data normalization.

In all cases, data were analyzed and represented as a heat map and/ or 2^(−ΔΔCT)^ using GraphBio [33] or GraphPad Prism version 9.3.1 for Mac, GraphPad Software, www.graphpad.com.

### Protein biology

#### Alkaline phosphatase staining

iPSCs were seeded and grown on geltrex-coated 24 × 24 mm glass coverslips in 6-well plates. iPSC colonies were washed with PBS and fixed in 4% paraformaldehyde (PFA) for 20 min. Colonies were washed three times with PBS and ALP-substrate staining solution (Abcam cat. Ab242287) was added as per manufacturer recommendations. Cells were incubated for 20 min in the dark at RT and then washed with PBS. Colony images were acquired and analyzed with the ECHO Rebel inverted microscope (ECHO).

#### Flow cytometry

1 × 10^6^ live cells were filtered through a 40 μm cell strainer (Thermo Fisher Scientific cat. 22363547) and fixed with 4% PFA for 20 min at RT. Upon fixation, cells were centrifuged at 700 g for 2 min and supernatant was removed. Cells were then permeabilized and stained using the BD Cytofix/Cytoperm Fixation/Permeabilization Solution Kit (BD Biosciences cat. 554714) as per manufactures instructions. Primary antibodies were incubated for 1 h and secondary antibodies for 30 min according to the dilutions in Additional file 1: Table S8. Cells were resuspended in fluorescence-activated cell sorting buffer (2% FCS, 2 mM EDTA in DPBS) and kept on ice until flow cytometry acquisition and analysis.

Isotype controls were used to accurately gate positive staining and data were acquired using the CytoFLEX S flow cytometer and analyzed using the CytExpert software (Beckman Coulter).

#### Immunohistochemistry

For 2D planar cell immunohistochemistry, cells were grown on coverslips and fixed with PFA as above. Cover slips were stored in PBS until staining.

3D suspension clusters were washed with PBS and fixed with 4% PFA for 30 min on ice. The PFA was removed and clusters were suspended in 1% low melting agarose within a silicone histology mold. The solidified agarose-cluster preparation was removed from the mold, placed in wax on a histology cassette for paraffin embedding and processing. Sections of 8 µm on glass slides were used. Slides were incubated for 40 min at 60 °C to melt the paraffin and allow cell adherence to the slides followed by rehydration. Slides underwent antigen retrieval in warmed citrate buffer (0.0126 M citric acid, Sigma cat. C-0759; 0.0874 M sodium citrate, Sigma cat. S-4641; pH 6.0) for a total of 20 min. Slides were then ready for staining.

Slides and cover slips were blocked for 1 h at RT with 5% normal donkey serum (Sigma cat. S30-M) in FoxP3 permeabilization buffer (Biolegend cat. 421402). Primary antibodies were diluted in FoxP3 permeabilization buffer as per Additional file 1: Table S8 and were incubated for 2 h at RT in a humid dark chamber. Slides and cover slips were washed 3 times with 0.1% Tween 20 in PBS followed by incubation with secondary antibodies diluted in FoxP3 permeabilization buffer for 40 min at RT in the dark. Slides and coverslips were washed 3 times in 0.1% Tween 20 in PBS prior to incubation with DAPI (Sigma, D1306) for 4 min at RT in the dark. Slides or cover slips were then washed with PBS and mounted with fluoromount-G (Thermo Fisher Scientific cat. 00–4958-02). Slides were visualized using the Zeiss Observer Z1 inverted fluorescence microscope, and images were processed using Zeiss software.

### Teratoma assay

Male immunocompromised SCID beige mice aged 16–18 weeks (Charles River Laboratories) were used. iPSCs were transplanted under the kidney capsule for 60-days (8 weeks), recovered, and assessed (n = 6 per group). For cells expanded in 2D planar, cells were lifted as per passaging protocols and transferred to a 15 mL conical tube with StemFlex supplemented with 10 μM RockI. Cells were aliquoted at 1 × 10^6^ cells per tube, which were centrifuged to remove media. Cells were combined with 15µL of matrigel (Sigma cat. CLS354277) and placed on ice. For 3D suspension preparations, cell clusters were collected from the bioreactor into microcentrifuge tubes and were ready for transplant (as clusters and without matrigel). In both cases, 3D expanded iPSC clusters or 2D expanded single cells embedded into matrigel were aspirated into polyethylene-50 tubing with a microsyringe. A left lateral paralumbar incision was made, and the left kidney was delivered. The kidney capsule was incised, and the cells were infused [34, 35]. Mice were anesthetized with 5% isoflurane. Buprenorphine (0.1 mg/kg subcutaneous) was administered for postoperative analgesia. Mice were assessed daily for humane end-points described by any mouse distress or change in physiologic condition. Throughout care, mice were housed within GM500 Mouse IVC Green Line cages in the Health Sciences Laboratory at the University of Alberta, in compliance with the Canadian Council on Animal Care guidelines.

On post-operative day 60, non-recovery nephrectomy was performed. Kidney cross sections were performed, fixed in 10% formalin, and paraffinized. 8 µm sections were stained with hematoxylin and eosin (H&E) or prepared for immunohistochemistry as above. H&E-stained slides were assessed by a board-certified pathologist.

### Statistical analysis

Normality testing was performed using the D’Agostino-Pearson normality test, which determined the need for non-parametric testing. Between-group comparisons were carried out using the nonparametric Mann–Whitney U test or Kruskal–Wallis test with the alpha value set at 0.05. Continuous values are presented as medians with interquartile ranges (IQR) and with discrete values presented as absolute values with percentages. All statistical analysis was completed using GraphPad Prism version 9.3.1 for Mac, GraphPad Software, www.graphpad.com.

## Results

### Generation of iPSC lines from human peripheral blood mononuclear cells 

**Fig. 1:**
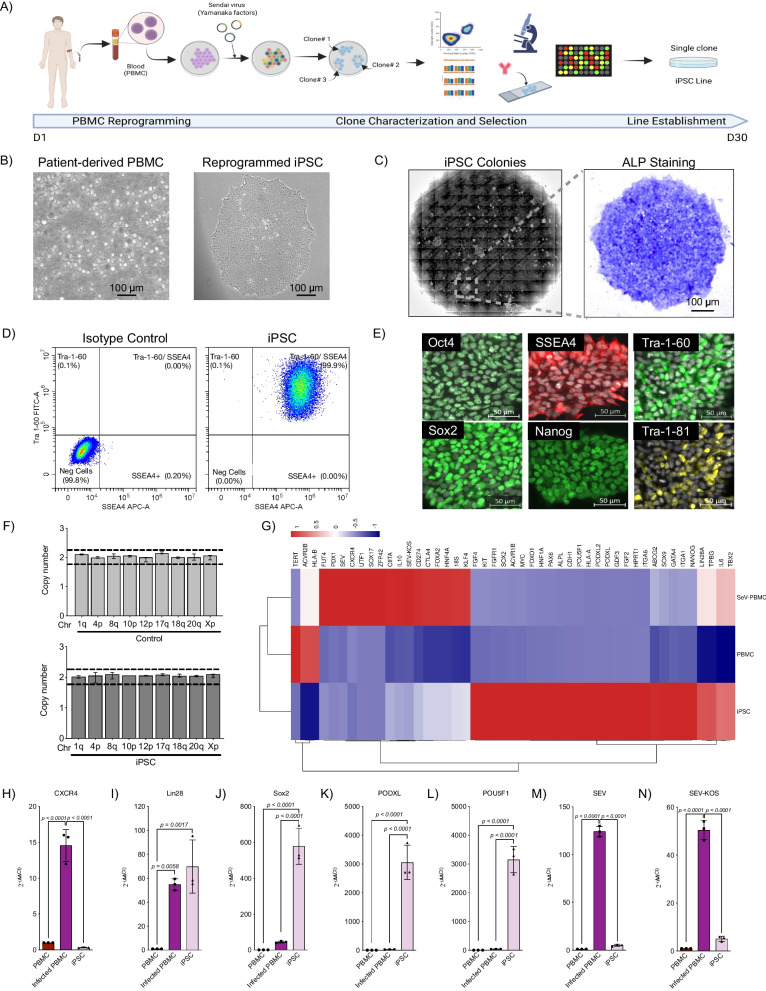
Establishment of iPSC line from human peripheral blood mononuclear cells. **A** Overview of processes for generating an induced pluripotent stem cell line including patient blood collection (day 1), peripheral blood mononuclear cell isolation, infection with Yamanaka factors, optimal clone selection, and iPSC line establishment (day 30). **B** Microscopy of peripheral blood mononuclear cells and established iPSCs. **C** Characterization of pluripotency of the established iPSC line using alkaline phosphatase (ALP) staining. **D** Flow cytometric analysis of the selected iPSC line with isotype control and characterization of Tra-1-60 and SSEA4 expression. **E** Immunohistochemistry of the established iPSC line with expression of Oct4, Sox2, SSEA4, and Tra-1–60. **F** Quantitative PCR evaluation of the established iPSC line frequently for genetic abnormalities within iPSCs comparing to commercially available control DNA (*n* = 9, 3 per iPSC line) **G** Genetic microarray results comparing established iPSCs to fibroblasts and peripheral blood mononuclear cells (*n* = 3, 1 per iPSC line) **H** Differential expression of *CXCR4*,  **I** *Lin28*, **J** *Sox2*, **K** *PODXL*, **L** *POU5F1*, **M** *SEV* and **N** *SEV-KOS* in PBMC, infected PBMC and iPSC (*n* = 3)

Following Sendai virus infection of human donor PBMCs, iPSC-like colonies were screened to select an optimal clone for iPSC line establishment (Fig. 1A). PBMCs grew independently with round shape, while iPSC-like colonies displayed compact cell-to-cell connections, rounded colony margins, and condensed nucleus with minimum cytoplasm (Fig. 1B). Manually picked and individually isolated clones (10–12 clones) were characterized at passage 3–5 according to current standards [22, 36]. Clones were assessed for expression of alkaline phosphatase (ALP) (Fig. 1C), Nanog, Tra-1–81, Sox2, Tra-1–60, and SSEA4 (Fig. 1D and Additional file 1: Fig. S1A, B). A single ALP-stained clone attaining 99.9% Tra-1–60^+^SSEA4^+^ and 98.3% Sox2^+^Nanog^+^ was selected for iPSC line establishment (Additional file 1: Fig. S1A). Immunohistochemistry demonstrated the selected clone to be positive for Oct4, Sox2, SSEA4, Nanog, Tra-1-81, and Tra-1-60 (Fig. 1E). The clone’s lack of duplications or deletions at the 8 most commonly mutated regions within iPSC lines was demonstrated by lack of alteration in copy number (Fig. 1F) [37, 38]. Upon establishment of the iPSC line, genomic profiling of 48 key human pluripotency targeted genes using TaqMan low density array cards displayed downregulation of somatic cell markers, like *Sox17* and *IL6*, while observing pronounced upregulation of pluripotency marker expression such as *Lin28*, *Sox2* and *PODXL* (Fig. 1G-L). Furthermore, *SEV* and *SEV-KOS* levels were identical to uninfected PBMC levels, which do not express *SEV* and *SEV-KOS* genes, ensuring lack of Sendai virus host-genome integration (Fig. 1M-N). PCR of the established iPSC cell lines at passage 10 using primers specific for the amplification of the Sendai virus further confirmed lack of Sendai viral vector integration (Additional file 1: Fig. S1C). PCR of the genetic material present in the supernatant confirmed lack of mycoplasma contamination (Additional file 1: Fig. S1C). These results demonstrate efficient reprogramming of PBMCs into iPSCs. The process was repeated to generate iPSC lines from four healthy volunteers.

### 3D suspension condition supports increased iPSC expansion 

**Fig. 2:**
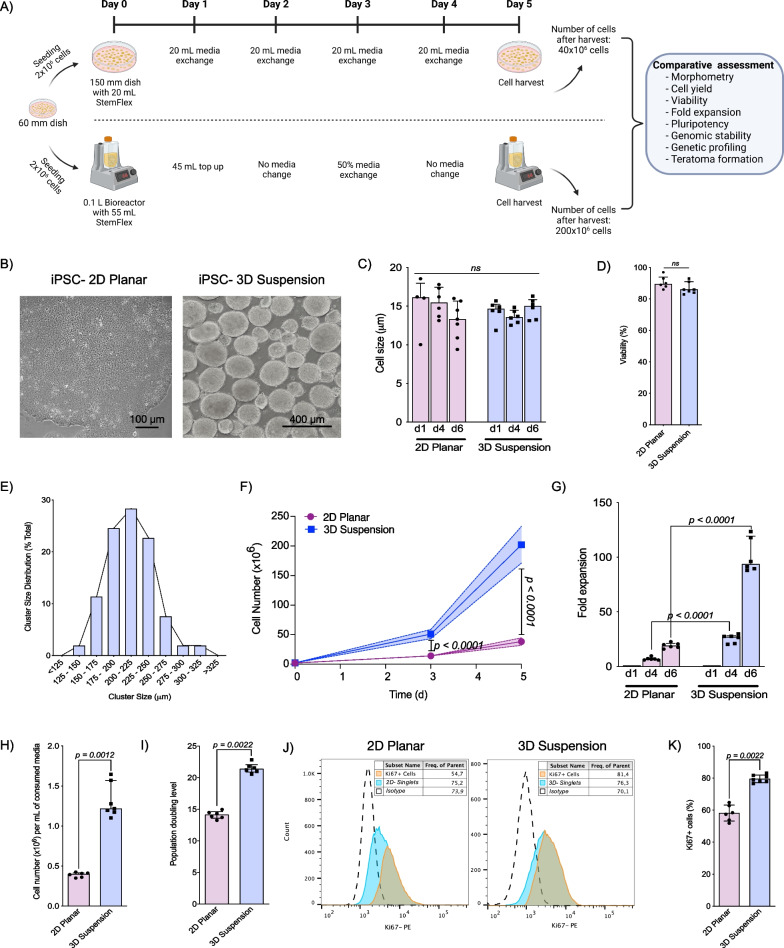
Evaluation of iPSCs expanded in two-dimensional planar (2D) and three-dimensional suspension (3D) cell culture. **A** Schematic representation of the expansion protocols for 2D and 3D suspension conditions with summary of techniques used to compare cells. **B** Morphology of cells expanded in 2D planar cell culture and 3D suspension expansion within Vertical-Wheel® bioreactors **C** Cell size following 3D cluster dissociation and 2D cell passaging on days 0, 3, and 5 of expansion (*n* = 6). **D** Cell viability following 5 days of cell expansion comparing 2D and 3D conditions (*n* = 6). **E** Cluster size for cells grown in 3D conditions with frequency of clusters characterized (*n* = 3). **F** Absolute cell number expansion using 2D and 3D cell culture (*n* = 6). **G** Fold expansion following 3 and 5 days of cell expansion in 2D and 3D cell culture (*n* = 6). **H** Cell expansion per milliliter of consumed media following 5 days of cell expansion in 2D and 3D cell culture (*n* = 6 per group). **I** Population doubling level for cells expanded in 2D and 3D conditions (*n* = 6 per group). **J** Representation of the gating strategy followed for the quantification of Ki67^+^ cells in 2D and 3D conditions. **K** Ki67 expression of cells expanded in 2D and 3D conditions (*n* = 6 per group)

**Fig. 3:**
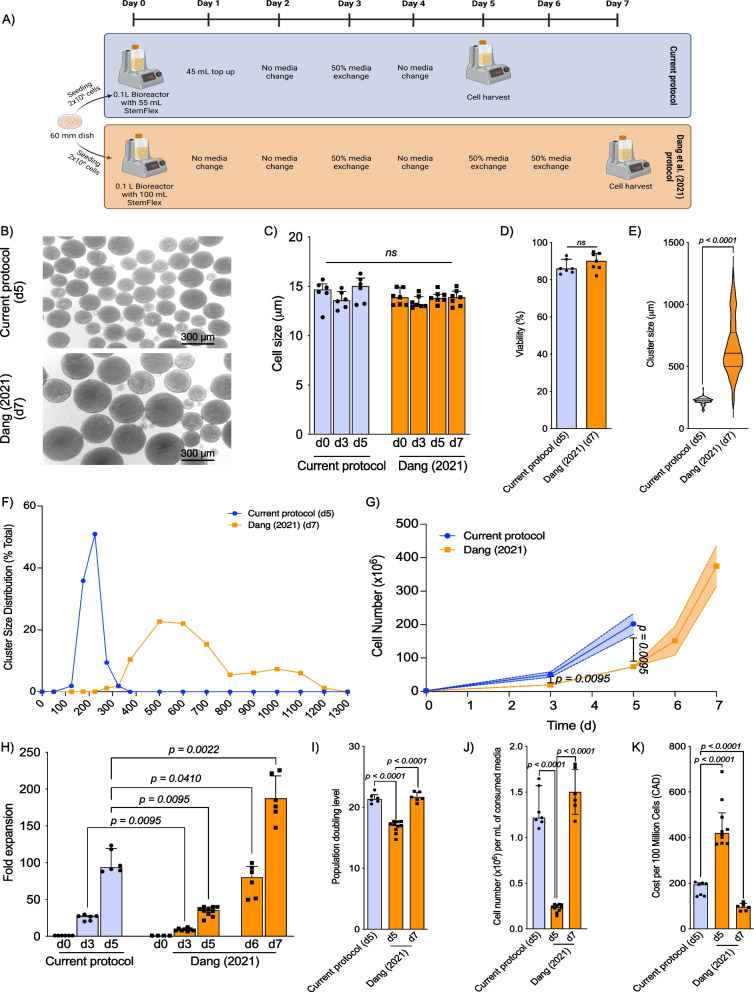
iPSC expansion comparison within Vertical-Wheel® bioreactors using the current protocol and replication of Dang et al. [28] protocols. **A** Schematic representation of the current protocol and the replicated Dang et al. [28] expansion protocol. **B** Cluster morphology at termination of iPSC expansion protocol using our current protocol or replicated Dang et al. [28] protocols. **C** Cell size following 3D cluster dissociation after expansion using the current protocol and replicated Dang et al. [28] protocol on days 0, 3, and 5 of expansion (*n* = 6). **D** Cell viability following 5 days of cell expansion comparing the current protocol and replicated Dang et al. [28] protocol (*n* = 6). **E** Cluster size for cells grown using the current protocol and replicated Dang et al. [28] protocol with frequency of clusters characterized (*n* = 6). **F** Cluster size distribution at termination of iPSC expansion protocol using the current protocol and replicated Dang et al. [28] protocol. **G** Absolute cell number expansion using the current protocol and replicated Dang et al. [28] protocol (*n* = 6). **H** Fold expansion following 3 and 5 days of cell expansion using the current protocol and 3, 5, 6 and 7 days of cell expansion using the replicated Dang et al. [28] protocol (*n* = 6). **I** Population doubling level for cells expanded using the current protocol and replicated Dang et al. [28] protocol (*n* = 6 per group). **J** Cell expansion per milliliter of consumed media following 5 days of cell expansion using the current protocol and 5 and 7 days of cell expansion using the replicated Dang et al. [28] protocol (*n* = 6 per group). **K** Cost of producing 100 × 10^6^ cells in 2023 Canadian Dollars following 5 days or 5 and 7 days of cell expansion using the current protocol and replicated Dang et al. [28] protocol (*n* = 6 per group)

Following iPSC line establishment, 2 × 10^6^ cells from 60 mm dishes were cultured in either 2D planar or 3D suspension conditions for a 5-day expansion cycle followed by cell harvest and head-to-head comparative assessment (Fig. 2A). Cells expanded in 2D planar conditions grouped tightly together to form compact colonies with well-delineated borders, which generated a monolayer sheet of cells upon confluency. Comparatively, cells grown in 3D suspension formed tight clusters that grew outwards in all directions with a central cavity (similar to epiblast structure during embryo formation), allowing cell microstructure support from nearby cells (Fig. 2B). 2D planar and 3D suspension expanded cells demonstrated no difference in single cell size (Fig. 2C) or viability (2D: 89.5% [IQR 6.5] vs. 3D: 86.0% [IQR 7.0], *p* = 0.75; Fig. 2D) throughout expansion. Notably, when 3D expanded cells were dissociated and re-plated they acquired identical architecture to 2D expanded cells; they generated compact colonies with delineated borders. The majority of iPSC clusters in 3D suspension were 175–250 µm (range: 125–324 μm) by the end of the 5-day expansion cycle (Fig. 2E). Following 3 days of expansion, comparatively more cells were generated using 3D suspension (2D: 13.5 × 10^6^ [IQR 3.7 × 10^6^] vs. 3D: 54.2 × 10^6^ [IQR 14.7 × 10^6^], *p* < 0.0001), with even greater expansion in 3D suspension after 5 days (2D: 40.1 × 10^6^ [IQR 8.5 × 10^6^] vs. 3D: 187.5 × 10^6^ [IQR 60.4 × 10^6^], *p* < 0.0001; Fig. 2F). Fold expansion was significantly greater under 3D suspension condition both on day 3 (2D: 6.7-fold [IQR 1.9] vs. 3D: 27.1-fold [IQR 7.4], *p* < 0.0001) and day 5 (2D: 19.1-fold [IQR 4.0] vs. 3D: 93.8-fold [IQR 30.2]; *p* < 0.0001; Fig. 2G). Results were similar for all iPSC lines (n = 4, Additional file 1: Fig. S2). At day 5, the number of cells generated per consumed mL of media was significantly higher for cells grown in 3D suspension compared to 2D planar conditions (2D: 4.0 × 10^5^ cells/ml [IQR 5.7 × 10^4^ cells/ml] vs. 3D: 1.2 × 10^6^ cells/ml [IQR 4.0 × 10^5^ cells/ml], *p* < 0.0001; Fig. 2H). Extrapolating from these data, the generation of 1 × 10^6^ iPSCs would cost significantly less in 3D suspension compared to 2D planar (2D: $417.7 [IQR $270.8] vs. 3D: $196.0 [IQR $58.9], *p* < 0.0001; 2022 Canadian Dollars; Fig. 3H). Similarly, the population doubling level was significantly higher in 3D suspension condition (2D: 14.1 [IQR 1.0] vs. 3D: 21.4 [IQR 1.19], *p* = 0.0022; Fig. 2I). Increased population doubling level was confirmed by a significantly increased percentage of proliferative (Ki67^+^) cells in 3D suspension condition on day 5 (2D: 57.4% [IQR 10.9%] vs. 3D: 76.6% [IQR 6.5%], *p* = 0.0022, Fig. 2J, K).

Media collected during 2D planar and 3D suspension iPSC expansion showed no statistically significant difference in pH or glucose, lactate or glutamine concentrations (Additional file 1: Fig. S3A–H). Supernatant from iPSCs expanded in 3D suspension had significantly lower concentrations of lactate dehydrogenase and ammonia than those from iPSCs expanded in 2D planar condition (Additional file 1: Fig. S3I–L).

28In addition, we compared our expansion protocol (current protocol from hereon) to the previously published 7-day 3D expansion protocol within Vertical-Wheel® bioreactors by Dang et al. [28]. We performed parallel experiments using both protocols; to reduce inter-protocol variability, we cultured cells using StemFlex rather than the modified B8 media initially reported by Dang et al. [28]. The primary differences between the two protocols include inoculation procedure (Current protocol: 2 × 10^6^ cells into 55.0 mL of StemFlex with 10 μM RockI on day 0 then top up to 100 mL on day 1; Dang et al. [28]: 2 × 10^6^ cells into 100 mL of StemFlex with 10 μM RockI on day 0) and feeding regime (Current protocol: 50% media replacement on d3 and harvest on d5; Dang et al. [28]: 50% media replacement on day 3, day 5 and day 6, and harvest on day7) (Fig. 3A). Regardless of the protocol used, cells were cultured with a constant rotational speed of 60 rpm. In both cases, cells formed tight clusters that grew outwards in all directions with a central cavity allowing cell microstructure support from nearby cells (Fig. 3B). Single cell size and viability were not different between cells from each protocol (Fig. 3C and D). However, replication of the protocol [28] produced iPSC clusters with median size of 607.7 μm [IQR 271.1 μm] after 7 days compared to 229.3 μm (IQR 10.8 μm] after 5 days of expansion using our protocol (*p* < 0.001, Fig. 3E); furthermore, the cluster size distribution was wider following the Dang protocol (Fig. 3F). Additionally, comparatively more cells were generated using our protocol following 3 days of expansion (Current protocol: 54.2 × 10^6^ [IQR 14.7 × 10^6^] vs Dang et al. [28]: 18.96 × 10^6^ [IQR 6.83 × 10^6^], *p* = 0.0095) and after 5 days (Current protocol: 187.5 × 10^6^ [IQR 60.4 × 10^6^] vs Dang et al. [28]: 97 × 10^6^ [IQR 18.17 × 10^6^], *p* = 0.0095) (Fig. 3G). Similarly, fold expansion was significantly greater following this study’s 3D suspension expansion protocol both on day 3 (Current protocol: 27.1-fold [IQR 7.4] vs Dang et al. [28]: 9.48-fol [IQR 4.5], *p* = 0.0095) and day 5 (Current protocol: 93.8-fold [IQR 30.2] vs Dang et al. [28]: 48.5 [IQR 7.7], *p* = 0.0095; Fig. 3H). Reduced fold expansion after 5 days of the Dang et al. [28] expansion protocol is a result of reduced population doubling (Current protocol: 21.4 [IQR 1.2] vs Dang et al. [28]: 17.1 [IQR 2.9], *p* < 0.0001; Fig. 3I) which resulted in decreased cell number per mL of consumed media (Current protocol: 1.2 × 10^6^ cells/ml [IQR 4.0 × 10^5^] cells/ml vs Dang et al. [28]: 0.65 × 10^6^ cells/ml [IQR 1.1 × 10^5^] cells/ml, *p* = 0.0061; Fig. 3J) and increased cost per 100 million cells (Current protocol: $196.0 [IQR $58.9] vs Dang et al. [28]: $308.0 [IQR $46.37], *p* = 0.0061; Fig. 3K). However, when the Dang protocol is continued until day 7, a significantly greater number of cells are generated (361.59 × 10^6^ [IQR 116.81 × 10^6^], and a higher fold expansion is achieved (Current protocol: 93.8-fold [IQR 30.2] vs Dang et al. [28]: 180.8 [IQR 78.11], *p* = 0.0022), compared to our 5-day protocol (Fig. 3G, H). This leads to a reduced cost per 100 million cells using the complete 7-day Dang protocol compared to our protocol (Fig. 3K). Interestingly, despite reduced population doubling level on day 5, there were no differences in the population doubling levels between protocols upon replication of the Dang et al. [28] protocol all the way to day 7 (Current protocol: 21.4 [IQR 1.19] vs Dang et al. [28]: 21.57 [IQR 2.03]; Fig. 3I).

### 3D suspension condition using Vertical-Wheel® bioreactors enables scalability

**Fig. 4:**
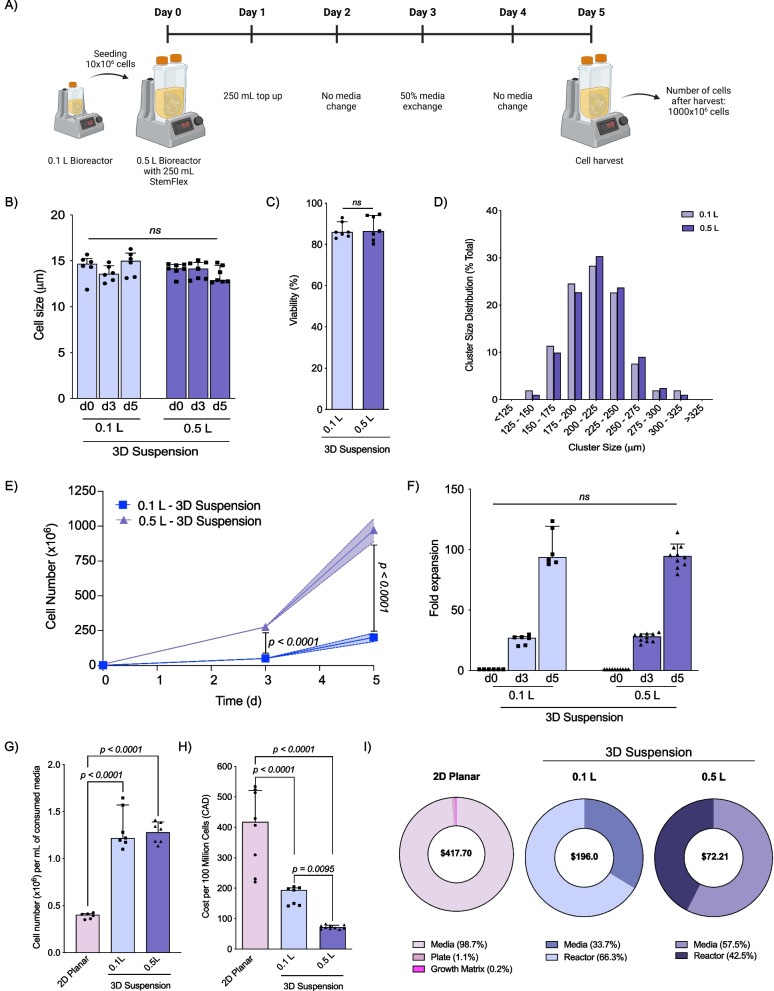
Comparison of expansion potential between 0.1 L and 0.5 L Vertical-Wheel® bioreactors. **A** Schematic representation of the expansion protocol used with 0.5 L Vertical-Wheel® bioreactor. **B** Cell size following 3D cluster dissociation from 0.1 L and 0.5 L Vertical-Wheel® bioreactor on days 0, 3, and 5 of expansion (*n* = 6). **C** Cell viability following 5 days of cell expansion comparing 2D and 3D conditions (*n* = 6). **D** Cluster size distribution for clusters grown in 0.1 L and 0.5 L Vertical-Wheel® bioreactors with frequency of clusters characterized (*n* = 3). **E** Absolute cell number expansion using 0.1 L and 0.5 L Vertical-Wheel® bioreactors (*n* = 6). **F** Fold expansion following 3 and 5 days of cell expansion in 0.1 L and 0.5 L Vertical-Wheel® bioreactors (*n* = 6). **G** Cell expansion per milliliter of consumed media following 5 days of cell expansion in 0.1 L and 0.5 L Vertical-Wheel® bioreactors and 2D planar conditions (*n* = 6). **H** Cost associated to the generation of 100 × 10^6^ cells following 2D planar or 3D suspension conditions using 0.1 L or 0.5 L Vertical-Wheel® bioreactors. **I** Representation of cost associated to media, plate and growth matrix or reactor

Following one passage of 3D suspension culture, iPSC clusters were dissociated and 10 × 10^6^ cells from 0.1 L Vertical-Wheel® bioreactors were seeded into 0.5 L Vertical-Wheel® bioreactor with a constant rotational speed of 60 rpm and cultured for a 5-day expansion cycle (Fig. 4A). 3D suspension expanded cells in 0.1 L or 0.5 L Vertical-Wheel® bioreactors demonstrated no difference in single cell size (Fig. 4B) or viability (0.1 L: 86.0% [IQR 7.0] vs. 0.5 L: 86.5% [IQR 12.0], *p* = 0.5979); Fig. 4C) throughout expansion. Volume capacity of the Vertical-Wheel® bioreactor did not alter the cluster size distribution; most iPSC clusters in 3D suspension were 175–250 µm (range: 125–324 μm) by the end of the 5-day expansion cycle regardless of the size of bioreactor used (Fig. 4D). Following 3 days of expansion, comparatively more cells were generated using 0.5 L Vertical-Wheel® bioreactors (0.1 L: 54.2 × 10^6^ [IQR 14.7 × 10^6^] vs. 0.5 L: 272.7 × 10^6^ [IQR 30.4 × 10^6^], *p* < 0.0001), with even greater expansion in 3D suspension after 5 days (0.1 L: 187.5 × 10^6^ [IQR 60.4 × 10^6^] vs. 0.5 L: 997.1 [IQR 164.3], *p* < 0.0001; Fig. 4E). Scale-up to 0.5 L Vertical-Wheel® bioreactor did not affect fold expansion at day 3 (0. 1L: 27.1-fold [IQR 7.4] vs. 0.5 L: 28.3-fold [IQR 10.3], p = 0.3676) or day 5 (0.1 L: 93.8-fold [IQR 30.2]; vs. 0.5 L: 94.5 [IQR 34.7], *p* = 0.4923; Fig. 4F). The number of cells generated per consumed mL of media at day 5 was significantly higher for cells grown in 3D suspension, regardless of the size of Vertical-Wheel® bioreactor used, compared to 2D planar condition (2D: 4.0 × 10^5^ cells/ml [IQR 5.7 × 10^4^ cells/ml] vs. 0.1 L: 1.2 × 10^6^ cells/ml [IQR 4.0 × 10^5^ cells/ml], *p* < 0.0001; 0.5 L: 1.3 × 10^6^ cells/ml [IQR 2.7 × 10^5^ cells/ml], *p* < 0.0001; Fig. 4G). Scale-up did not affect the number of cells generated per consumed mL of media at day 3 or 5. Extrapolating from these data, the generation of 1 × 10^6^ iPSCs would cost significantly less in 0.5 L Vertical-Wheel® bioreactors compared to suspension culture in 0.1 L Vertical-Wheel® bioreactors or 2D planar culture (2D: $417.7 [IQR $270.8] vs. 0.5 L: $70.4 [IQR $ 18.4], *p* < 0.0001; 0.1 L: $196.0 [IQR $58.9], *p* < 0.0001; 2022 Canadian Dollars; Fig. 4H). The cost breakdown and comparison between 2D planar and 3D suspension conditions using 0.1 L and 0.5 L Vertical-Wheel® bioreactors is shown in Fig. 4I.

### 3D suspension condition promotes superior pluripotency phenotype

**Fig. 5:**
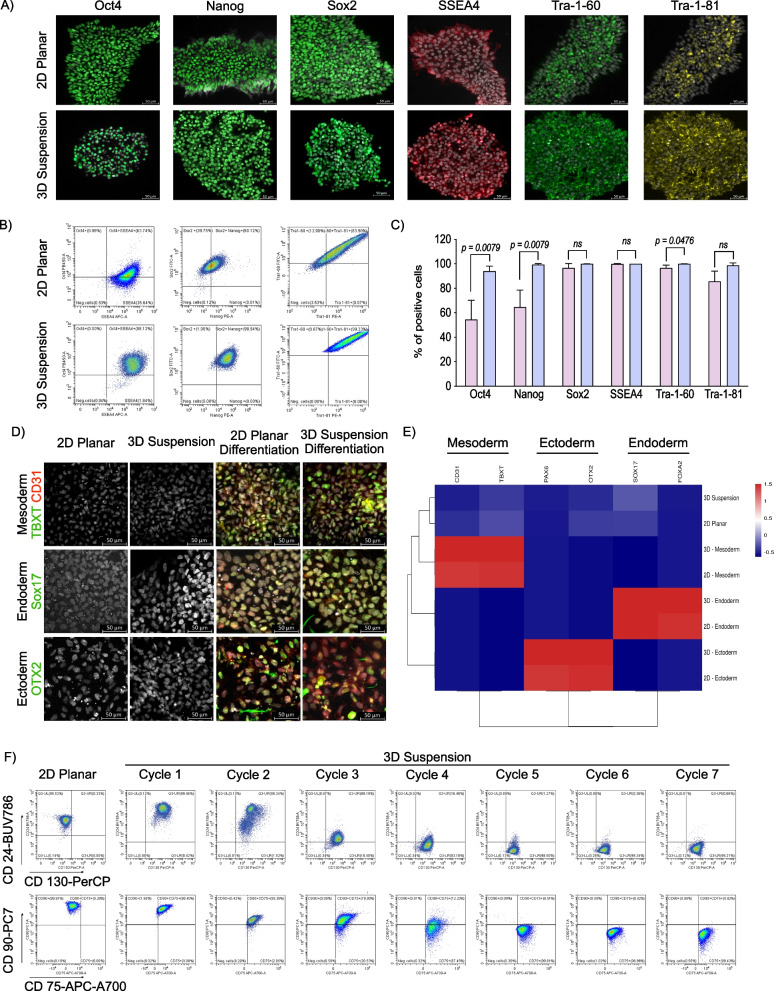
Comparative quantification of pluripotency marker expression. **A** Immunohistochemistry evaluation of pluripotency marker expression for cells expanded using 2D and 3D cell culture. **B** Flow cytometric analysis to quantify pluripotency marker expression of iPSCs expanded in 2D and 3D conditions (*n* = 6 per group). Single stained results for the right panel can be found in Additional file 1: Fig. S1B. **C** Quantification of pluripotency marker expression of iPSCs expanded in 2D and 3D conditions (*n* = 6 per group). **D** Flow cytometric analysis to show the expression of CD24, CD130, CD90 and CD75 upon the transition of iPSCs from 2 to 3D conditions (*n* = 3 per group). **E** Comparison of the expression of key mesoderm, ectoderm and endoderm lineage associated genes among iPSCs cultured in 2D and 3D conditions as well as differentiated cells cultured under 2D and 3D conditions. **F** Flow cytometric analysis of the transition of primed to naïve cells. Single cells were selected and examined for the expression of CD24, CD130, CD90 and CD75

Qualitative assessment of protein level pluripotency marker expression by cells expanded under both conditions was completed using immunohistochemistry and demonstrated that both 2D planar and 3D suspension expanded iPSCs displayed classical markers of pluripotency including Oct4, Nanog, SSEA4, Sox2, Tra-1-60, and Tra-1-81 (Fig. 5A). Quantification of pluripotency markers using flow cytometry demonstrated that significantly more 3D expanded cells co-express Oct4, Nanog, and Sox2 (2D: 52.5% [IQR 5.6%] vs. 3D: 94.3% [IQR 1.4%], *p* = 0.0079, Fig. 5B and C), with more cells expanded in 2D planar culture failing to co-express Tra-1–60 and Tra-1–81 (2D: 3.3% [IQR 0.9%] vs. 3D: 0% [IQR 0%], *p* = 0.0476, Table 1). Complete gating strategies and quantification can be found in Additional file 1: Fig. S1 and Table 1 respectively.

**Table 1:** Pluripotency marker expression of 2D and 3D expanded cells

	2D planar	3D Suspension	p-value
Tra-1-60^−^ Tra-1-81^−^	3.31% (IQR 0.95%)	0.00% (IQR 0.00%)	0.0476
Tra-1-60^−^ Tra-1-81^+^	0.08% (IQR 0.04%)	0.00% (IQR 0.00%)	0.0476
Tra-1-60^+^ Tra-1-81^−^	11.05% (IQR 2.83%)	1.20% (IQR 0.87%)	0.0952
Tra-1-60^+^ Tra-1-81^+^	85.57% (IQR 3.75%)	98.80% (IQR 0.87%)	0.0952
Oct4^−^ Nanog^−^ Sox2^−^	5.47% (IQR 2.21%)	0.09% (IQR 0.09%)	0.0476
Oct4^−^ Nanog^−^ Sox2^+^	26.92% (IQR 5.98%)	1.13% (IQR 0.42%)	0.0079
Oct4^−^ Nanog^+^ Sox2^−^	0.09% (IQR 0.04%)	0.01% (IQR 0.01%)	0.0873
Oct4^−^ Nanog^+^ Sox2^+^	5.89% (IQR 0.85%)	3.95% (IQR 1.30%)	0.3095
Oct4^+^ Nanog^−^ Sox2^−^	0.27% (IQR 0.16%)	0.01% (IQR 0.01%)	0.0873
Oct4^+^ Nanog^−^ Sox2^+^	6.46% (IQR 2.19%)	0.25% (IQR 0.08%)	0.0079
Oct4^+^ Nanog^+^ Sox2^−^	0.32% (IQR 0.15%)	0.00% (IQR 0.00%)	0.0476
Oct4^+^ Nanog^+^ Sox2^+^	52.45% (IQR 5.61%)	94.25% (IQR 1.40%)	0.0079

Quantitative pluripotency marker expression on day 5 characterized by flow cytometry of cells expanded in 2D planar and 3D suspension conditions with percent of total cells and *p*-value

To evaluate cells from both conditions for spontaneous trilineage differentiation cells were stained for ectoderm, endoderm and mesoderm markers (Fig. 5D) and transcripts for these markers were evaluated. Immunochemistry assessment showed that neither 2D planar or 3D suspension expanded cells expressed markers for ectoderm (Pax6 and OTX2), endoderm (Sox17 and FoxA2) or mesoderm (CD31 and TBXT) (Fig. 5D). Similarly, qRT-PCR demonstrated that both 2D planar and 3D suspension cells did not demonstrate expression of trilineage transcripts (Fig. 5E and Additional file 1: Fig. S4B–D). Additionally, embryoid bodies generated from 2D planar and 3D suspension expanded cells did not express trilineage markers on immunohistochemistry or within their transcripts (Additional file 1: Fig. S4A–D). Assessment of pluripotency marker expression at transcript level can be found in Additional file 1: Fig. S4E–K. Although no spontaneous differentiation was noted, both 2D planar and 3D suspension cells were capable of trilineage differentiation (Fig. 5D–E).

To study the effect of 2D planar and 3D suspension expansion on pluripotency status, we evaluated cells for primed (CD24 and CD90) and naïve (CD130 and CD75) pluripotency phenotype markers using flow cytometry. The differential expression of these markers has previously been reviewed by Collier et al. [39] demonstrating these to represent the most specific markers for naïve and primed pluripotency phenotypes. Under 2D planar conditions, 98.5% (IQR 1.0%) and 99.6% (IQR 0.3%) of cells were CD24^+^CD130^−^ and CD90^+^CD75^−^, respectively (Fig. 5F). Following the first 3D suspension passage, iPSCs began expressing naïve iPSC markers and became CD24^+^CD130^+^ and CD90^+^CD75^+^. Gradual transition of iPSCs from primed (CD24^+^CD130^−^ and CD90^+^CD75^−^) to naïve (CD24^−^CD130^+^ and CD90^−^CD75^+^) occurred with continued 3D suspension culture, with 98.40% (IQR 1.14] of cells being CD24^−^CD130^+^ and 99.10% (IQR 0.75%) being CD90^−^CD75^+^ after 5 passages under 3D suspension conditions (Fig. 5F). Complete flow cytometric analysis including pluripotency and primed/naïve marker expression upon 10 subsequent passages in 2D planar or 3D suspension conditions can be found in Additional file 1: Fig. S5. Due to these findings, further 2D planar and 3D suspension comparisons were made on cells expanded using each condition for at least 5 passages.

### 3D suspension conditions induce transcriptional changes without promoting copy number variations

**Fig. 6:**
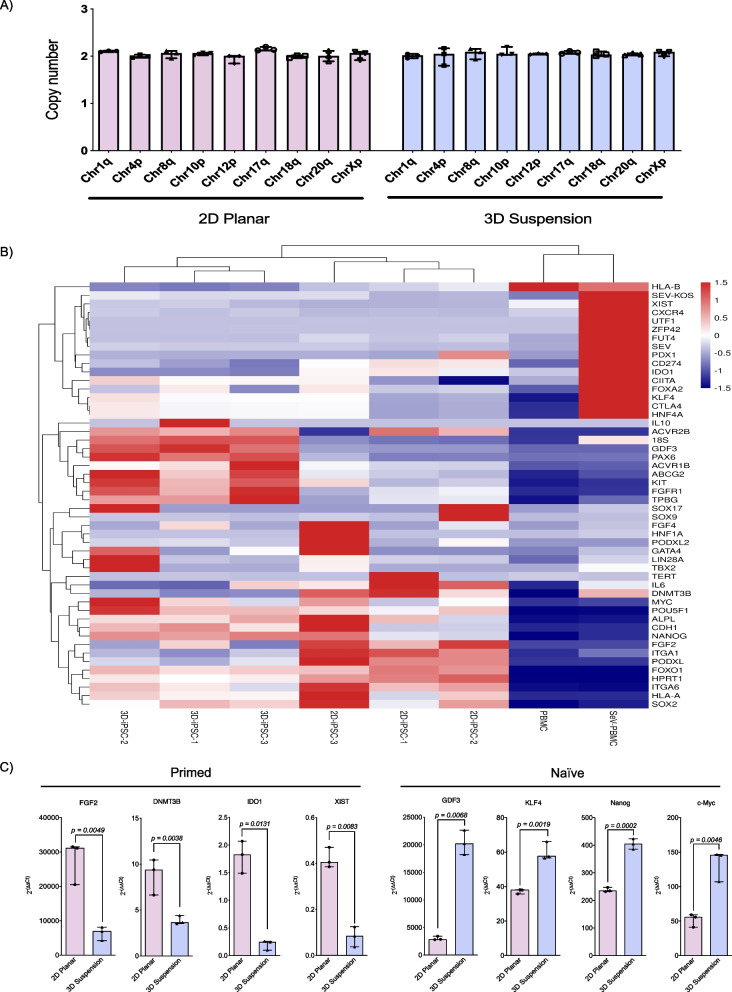
Comparative assessment of chromosomal stability and gene expression. **A** Quantitative PCR evaluation of the established iPSC line frequently for genetic abnormalities within cells expanded in 2D and 3D conditions (*n* = 3 per group). **B** Heat map showcasing differential gene expression between cells expanded in 2D and 3D conditions. **C** Differential expression in 2D and 3D of primed markers *FGF2*, *DNMT3B*, *IDO1* and *XIST*, and naïve markers *GDF3*, *KLF4*, *Nanog* and *c-Myc* (all *n* = 3 per group)

Genetic analysis evaluating the most frequently mutated genomic locations during iPSC expansion showed that both 2D planar and 3D suspension conditions did not demonstrate any deletions or duplications (Fig. 6A). Transcriptomic analysis of pluripotency genes showed that iPSCs expanded under both conditions had upregulated pluripotency gene transcription, including *POU5F1*, *Nanog* and *Sox2*, compared to the patient-derived PBMCs and the PBMCs 4-days after Sendai virus infection (Fig. 6B). More importantly, under 2D planar and 3D suspension conditions biological replicates clustered independently from each other, highlighting the effect that culture conditions have on iPSC transcriptomics. 2D planar cells transcribed significantly more *FGF2* (2D: 31,114.0 [IQR 11024.0] vs. 3D: 6909.0 [IQR 3901.0], *p* = 0.0049), *DNMT3B* (2D: 9.3 [IQR 3.8] vs. 3D: 3.7 [IQR 0.9], *p* = 0.0038)*, ID01* (2D: 1.8 [IQR 0.6] vs. 3D: 0.24 [IQR 0.2], *p* = 0.0131)*,* and *XIST* (2D: 0.4 [IQR 0.08] vs. 3D: 0.08 [IQR 0.08], *p* = 0.0083) than 3D suspension cells, while 3D suspension cells transcribed significantly more *GDF3* (2D: 2852.0 [IQR 610.0] vs. 3D: 20,211.0 [IQR 4420.0], *p* = 0.0068), *KLF4* (2D: 38.0 [IQR 2.4] vs. 3D: 57.6 [IQR 9.6], *p* = 0.0019), *Nanog* (2D: 234.7 [IQR 15.2] vs. 3D: 405.0 [IQR 37.9], *p* = 0.0002) and *c-Myc* (2D: 55.8 [IQR 18.1] vs. 3D: 145.5 [IQR 39.6], *p* = 0.0046) (Fig. 6C).

### iPSCs expanded using 3D suspension conditions generate more mature teratomas with fewer proliferative cells 

**Fig. 7:**
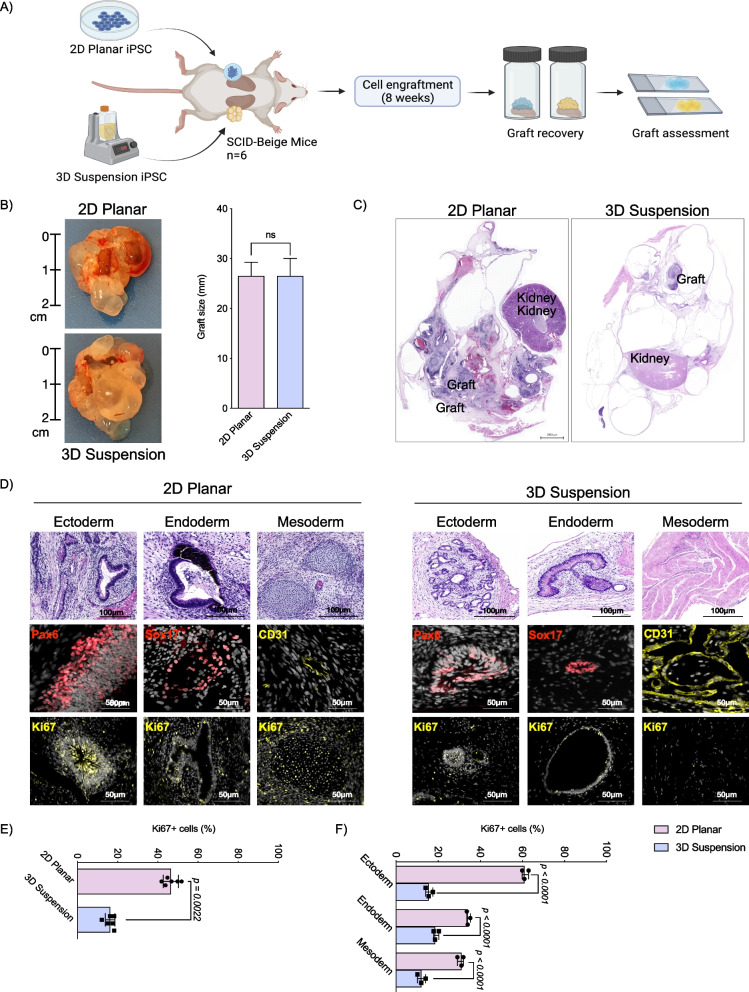
Teratoma formation assessment and comparison between iPSC expansion protocols. **A** Overview of process used for teratoma assay to characterize in vivo maturation of iPSCs (*n* = 6 per group). **B** Teratomas excised from transplanted mice with size comparison of grafts achieved from cells expanded in 2D planar and 3D suspension conditions. **C** Hematoxylin and eosin (H&E) staining of iPSC grafts transplanted into the renal subcapsular space following 2D and 3D cell expansion. **D** Histological characterization of iPSC-derived tumors demonstrating structures compatible with the three germ layers compatible with teratomas using H&E staining. Immunohistochemistry staining of iPSC grafts transplanted into the renal subcapsular space following 2D and 3D cell expansion with staining for PAX6 (ectoderm), SOX17 (endoderm), and CD31 (mesoderm) markers. Immunohistochemistry evaluation of Ki67 expression within 2D and 3D derived iPSCs with quantification of expression **E** and **F** All analyses represent *n* = 3 per group

To assess the impact of iPSC expansion conditions on in vivo pluripotency potential, cells from both conditions underwent renal subcapsular transplantation followed by graft harvest and assessment after 8 weeks of in vivo maturation (Fig. 7A). Cells from both conditions produced teratomas of equal size (2D: 26.5 mm [IQR 7.5 mm] vs. 3D: 26.5 mm [IQR 6.5 mm], *p* = 0.85; Fig. 7B). However, morphology of the grafts generated by 2D planar and 3D suspension conditions differed, with 2D planar cells generating solid grafts and 3D suspension cells producing fluid-filled cystic grafts (Fig. 7C). Histological assessment following H&E staining of the recovered grafts showed that all grafts had representative tissues from the three germ layer lineages (i.e., teratomas) (Fig. 7D). Graft characterization with immunohistochemistry demonstrated expression of PAX6 (ectoderm), SOX17 (endoderm), and CD31 (mesoderm) further confirming trilineage differentiation capacity (Fig. 7D). Teratomas from 3D expanded iPSCs assessed by a trained pathologist represented more mature ectoderm, mesoderm and endoderm tissues identified as exoskeletal stratified epithelial, mature muscle fibers and duct-glandular regions compared to the less mature tissues found in teratomas from 2D expanded cells visualized as neural rosettes, chondrocytes, and glandular tissue. Immunohistochemistry labeling for Ki67 demonstrated statistically fewer proliferative cells within the grafts generated from 3D suspension cells (2D: 45.3% [IQR 3.0%] vs. 3D: 16.7% [IQR 3.2%], *p* = 0.002; Fig. 7E) regardless of the germ layer evaluated (Fig. 7F).

## Discussion

This study presents a novel scalable iPSC expansion protocol using 3D suspension culture within Vertical-Wheel® bioreactors, achieving the greatest fold iPSC expansion in 5 days using these bioreactors reported to date. Cells expanded using this protocol acquire superior pluripotency phenotype compared to 2D planar expanded cells. Overall, as opposed to 2D planar culture, 3D suspension culture within Vertical-Wheel® bioreactors enables sufficient iPSC expansion for clinical implementation and offers a superior biomanufacturing process for economical, large volume generation of consistent, high-quality cell products that advances clinical implementation of iPSC-derived cell therapies.

Compared to previous iPSC expansion protocols (Table 2), our protocol offers superior cell expansion with optimal cell cluster size consistency. Earlier studies by Nogueira et al. [40] and Rodrigues et al. [41] expanded cells using single-use Vertical-Wheel® bioreactors and achieved < tenfold expansion over 5–6 days. More recently, two parallel studies by Borys et al. [27] and Dang et al*.* [28] demonstrated 30-fold iPSC expansion in 6 days and 62-fold iPSC expansion in 7 days, respectively, using Vertical-Wheel® bioreactors. Alternatively, Manstein et al. [42] demonstrated 70-fold iPSC expansion after 7 days using automated stirred tank bioreactors. However, evidence from Borys et al. [27] demonstrates caveats to horizontal-blade bioreactors due to high fluid force heterogeneity resulting in significant variation in cluster size compared to Vertical-Wheel® bioreactors. Replication of the Dang et al. [43] protocol using StemFlex media in our hands achieved 180.8-fold expansion but unfortunately generated large > 600 μm clusters. Previous literature demonstrates that cell clusters < 400 μm are optimal for differentiation and downstream cell product generation by limiting central core necrosis. Therefore, although the Dang et al. [28] protocol achieved substantial cell expansion, the updated 5-day protocol presented here has superior cell expansion at day 5 and achieves an optimal iPSC cluster size with a more consistent cluster size distribution that is better for subsequent differentiation. Overall, our modified expansion protocol demonstrates increased expansion (93.8-fold) over 5 days while maintaining consistent cell cluster sizes, indicative of a homogenous cell population. Reasons for this substantial increase in expansion using this protocol include use of StemFlex growth media, early dilution of RockI on day 2 of expansion, and optimally timed media changes.

**Table 2:** Comparison of recent published 3D suspension iPSC expansions studies in different bioreactor geometries

Reference	Year	iPSC Cell Lines Used	Media	Bioreactor Size and Type	Highest Fold Expansion/Days
Elanzew et al. [67]	2015	iLB-C-31f-r1	mTeSR1, E8	50 mL, Tube rotation (BioLevitator)	Fivefold/4 days
Haraguchi et al. [68]	2015	201B7, 253G1	mTeSR1	100 mL, Horizontal-blade (Integra Biosciences)	Tenfold/12 days
Badenes et al. [69]	2016	Gibco CD34 + derived	E8	50 mL, Horizontal-blade (StemSpan)	3.5-fold/10 days
Kropp et al. [26]	2016	hCBiPS2, hHSC_F1285T_iPS2	mTeSR1, E8	250 mL, Eight blade impeller (DASbox)	Sixfold/7 days
Meng et al. [70]	2017	4YA, 4YF	mTeSR1	100 mL, Horizontal-blade (NDS)	12- to 13-fold/5 days
Abecasis et al. [71]	2017	ChiPS C4, ChiPS C12, ChiPS C15, ChiPS C18, ChiPS C22	Cellartis DEF-CS Xeno-Free	200 mL, Trapezoid-paddle (DASGIP)	19-fold/4 days
Kwok et al. [72]	2018	AFiPS, FSiPS	mTeSR1, StemMACs iPS-Brew	125 mL, Horizontal-blade (Corning) 1000 mL, Horizontal-blade (Mobius)	16-fold/7 days
Rodrigues et al. [41]	2018	F002.1A.13, Gibco Human Episomal iPSC	E8	0.1 L, Vertical- Wheel® (PBS Biotech) 0.5 L, Vertical- Wheel® (PBS Biotech)	6.7-fold/6 days
Noguiera et al. [73]	2019	F002.1A.13, Gibco Human Episomal iPSC	mTeSR1, mTeSR3D	0.1 L, Vertical- Wheel® (PBS Biotech)	9.3-fold/5 days
Borys et al. [27]	2020	4YA	mTeSR1	0.1 L Vertical- Wheel® (PBS Biotech)	32-fold/6 days
Borys et al. [20]	2021	4YA	mTeSR1	0.1 L, Vertical- Wheel® (PBS Biotech) 0.5 L, Vertical- Wheel® (PBS Biotech)	32-fold/6 days
Manstein et al. [74]	2021	MHHi006-A, MHHi001-A, MHHi008-A	E8	250 mL, Eight blade impeller (DASbox)	70-fold/7 days
Dang et al. [28]	2021	4YA	mTeSR1, Modified B8	0.1 L mL, Vertical- Wheel® (PBS Biotech) 0.5 L, Vertical- Wheel® (PBS Biotech)	62-fold/7 days
Current protocol	2023	Healthy donor-derived cell lines	StemFlex	0.1 L mL, Vertical- Wheel® (PBS Biotech) 0.5 L, Vertical- Wheel® (PBS Biotech)	93.8-fold/5 days

Table adapted from Borys et al. [27]

This protocol’s modifications were based on several iterations and prior literature. First, StemFlex media was used as it contains thermostable FGF2; FGF2 has previously been described as a limiting factor for the expansion of human iPSCs [44]. FGF2 promotes phosphorylation of mitogen-activated protein kinases (MEK) and extracellular signal-regulated kinases (ERKs) to improve cell expansion, maintain cell pluripotency, reduce spontaneous differentiation, and direct cells toward a naïve pluripotency state [45, 46, 47, 48]. Replication of the Dang et al. [28] protocol using StemFlex resulted in higher fold expansion (180.8 vs. 62) than reported by their group using either mTeSR1 or modified B8 media, supporting the importance of thermostable FGF2 supplementation. However, considering that our protocol still achieves superior fold expansion at day 5 compared to the Dang et al. [28] protocol, it is likely that StemFlex alone is not the only factor contributing to this study’s results.

In addition to StemFlex use, early RockI elimination and media change at day 3 also contributed to the increased expansion potential of our protocol. Despite the role of RockI in early single cell survival and clustering, we diluted RockI on day 1 because it has also been described to reduce iPSC proliferation [49]. Similarly, media exchange on day 3 was utilized to ensure appropriate nutrient availability during the exponential growth phase. Assessment of the pH and metabolite concentration available in the culture supernatant during expansion ensured that reduced proliferation in 2D planar expanded iPSCs was not a result of unfavorable environment (pH or lactate) or decreased metabolite availability (glucose or glutamine). Interestingly, lactate dehydrogenase and ammonia concentration were increased in the 2D planar expansion media compared to the 3D suspension media, which suggests increased cell death and catabolism of amino acids in 2D planar conditions. Indeed, the combination of early RockI and ensuring adequate nutrient conditions for cells with only 2 media changes maximizes cell growth and economic benefits of our updated protocol.

Overall, it is likely that a combination of StemFlex media, early RockI removal, and optimally timed media exchanges promotes superior expansion demonstrated using this protocol. These changes optimized cell expansion and correlated with an increased percentage of proliferative (Ki67^+^) iPSCs and increased population doubling level. Practically, this means that although our protocol requires expensive bioreactors and uses more media, the total cost to generate 100 × 10^6^ iPSCs using 3D suspension condition is only 46.9% of the total cost compared to 2D planar condition—an attractive advantage for scalability. Furthermore, expansion using 0.5 L bioreactors offers even greater opportunity for cell expansion with increased cost savings and a much more scalable, robust process with fewer user interventions and the option of future automation to further increase repeatability.

In addition to demonstrating improved iPSC expansion, this study shows that 3D suspension expanded cells better express pluripotency markers and transition to a naïve pluripotency phenotype [50, 51, 52, 53, 54]. Stem cell culture with thermostable FGF2 has previously resulted in similar findings, potentially accounting for some of these results [45, 46, 47, 48]. Notably, the requirement for exogenous FGF2 supplementation should not be confused with reduced endogenous *FGF2* expression by 3D suspension expanded cells, as this represents independence from the MEK-ERK pathway, an important marker of naïve pluripotency phenotype [14, 55]. Regardless, both 2D planar and 3D suspension cells benefited from thermostable FGF2. Therefore, we hypothesize that phenotypic changes in 3D expanded cells occur due to growth within clusters that mimics epiblastic structures with supporting integrin microstructure provided by nearby cells [56, 57, 58]. This structural support allows akt1 activation downstream from FGF2, resulting in FGF2 independence [58]. Key advantages of the naïve pluripotency state have previously been well described including their improved capacity for differentiation [59, 60], and expansion with cell doubling time half that of their primed counterparts [59].

While cells expanded using each method were capable of in vitro trilineage differentiation, more thorough in vivo evaluation using the teratoma assay further supports the findings of a naïve phenotype of 3D suspension expanded cells. Teratomas generated from 3D suspension expanded cells had increased tissue maturity and decreased proliferation; others have demonstrated that lineage potential is protected in naïve cells, whereby naïve pluripotent cells can better differentiate and form mature teratoma tissue [59]. Clinically, increasingly mature teratomas have reduced risk of malignant transformation [61, 62]. Although limited studies exist evaluating the safety of iPSC therapies clinically, tumorigenic risk due to residual iPSCs remains a concern. As iPSC-derived cell therapies transition toward clinical applications, further optimization of expansion protocols is crucial to ensure scalability and safety of cell therapies. Assessment of the in vitro and in vivo characterization of cells expanded in 2D planar and 3D suspension conditions suggests that 3D suspension condition offers a potentially safer cell product with reduced risk of malignant and proliferative off-target growth originating from remnant non-differentiated iPSCs [63, 64].

These promising findings supporting iPSC expansion within Vertical-Wheel® bioreactors should be considered in the context of important limitations. First, while we provide one of the first direct comparisons of 2D planar to 3D suspension expansion techniques, both remain specific to the cell source origin, iPSC reprogramming techniques, matrices, bioreactors, and media used in this study. Previous studies have demonstrated that iPSC cell source does not affect subsequent expansion, however, limited studies have compared iPSC generation techniques (Sendai virus vs. other) [65]. It is possible that other 2D planar matrices could provide similarly high-quality iPSCs during expansion as those achieved in 3D suspension conditions; however, these matrices would confer an even greater cost limiting their translational applicability. Similarly, while we have demonstrated identical expansion capacity and cost savings by using fivefold larger 0.5 L bioreactors, further expansion using large commercial bioreactors (e.g., 3 L–15 L) remains untested and may not achieve similar efficacy due to differences in metabolism, hydrodynamic forces experienced by the cells, and the introduction of computer-controlled systems. Further evaluation of these factors during scale-up remain of importance for future investigation. However, the ability to control pH and dissolved oxygen at precise levels in the liquid in larger bioreactors would most likely offset any additional complexities during scale-up. Future studies evaluating larger bioreactors are needed, especially with regards to potential allogeneic cellular transplantation techniques whereby a single cell source could be expanded for many patients. Additionally, the mice used for teratoma assay were all male since female mice were allocated for breeding at the time of these experiments; sex has previously been shown to not affect the outcome of teratoma assays [66] but could potentially impact our results. Finally, this study offer data from 4 healthy donor iPSC lines and it remains unclear if patient factors will affect iPSC generation or expansion.

Considering ongoing optimization of iPSC-derived cell products, the importance of generating and exponentially expanding a reliable iPSC starting product should not be overlooked and will continue to become increasingly valuable as we approach broader clinical implementation. Despite these limitations, this study offers an updated iPSC expansion protocol achieving the greatest fold growth over 5 days reported to date using Vertical-Wheel® bioreactors. Not only does this protocol achieve superior cell expansion to previously reported 2D planar, and 3D suspension protocols using Vertical-Wheel® reactors, but yields iPSCs with superior pluripotency marker expression and a naïve pluripotency phenotype.

## Conclusions

This study demonstrates an improved iPSC expansion protocol using 3D Vertical-Wheel® bioreactors achieving almost 100-fold expansion over 5-days, representing the largest iPSC expansion reported to date. The ensuing 3D suspension expanded cell product appears to have improved expression of pluripotency markers with transition toward a naïve stem cell phenotype. Additionally, 3D suspension expanded cells are capable of trilineage differentiation and generate more mature and less proliferative teratomas. These results support application of 3D suspension techniques using Vertical-Wheel® bioreactors to efficiently produce high-quality iPSCs for subsequent differentiation into cell products for clinical implementation.

## Supplementary Information

**Additional file 1**. **Supplementary Figures. Figure S1.** Extended quality control performed on reprogrammed iPSC lines. **A** Gating strategy used for flow cytometric analysis of the selected iPSC line with isotype control. Briefly, forward and side scatter was used to identify the cell population and remove debris and other events of non-interest based on size and complexity. Width and height of cells was used to exclude the double or multiple cells from single cells. Single cells were selected for further analysis and examined for the expression of Oct4, SSEA4, Nanog and Sox2. Isotype controls were used to accurately gate positive staining and data were acquired using the CytoFLEX S flow cytometer and analysed using the CytExpert software (Beckman Coulter). **B** Gating strategy for cytometric analysis of Tra-1-60 and Tra-1-81 with single stain results. **C** Clearance of reprogramming vectors and lack of mycoplasma contamination. To test the absence of the Sendai reprogramming vectors a PCR that detects the Sendai virus genome and the transgenes, was used. PCR products were analysed by 1% agarose gel electrophoresis. iPSCs were tested for the expression of Sev, KOS, KLF4, and c-Myc with β-actin as an internal control. Infected PBMC were used as positive control for transgene presence while un-infected PBMC were used as negative control. Similarly, Mycoplasma PCR Detection Kit was used to detect contamination by 200+ strains of Mycoplasmas. This kit includes a positive Mycoplasma control and water was used as negative control. Full-length blots/gels are presented. **Figure S2.** Expansion and evaluation of four iPSC lines expanded in 2D planar and 3D suspension cell culture. **A** Cell size following 3D suspension cluster dissociation and 2D cell passaging on days 0, 3, and 5 of expansion of three iPSC lines. **B** Absolute cell number expansion using 2D planar and 3D suspension cell culture of iPSC line 1, **C** iPSC line 2, **D** iPSC line 3 and **E** iPSC line 4. **F** Fold expansion following 3 and 5 days of cell expansion in 2D planar and 3D suspension cell culture of four iPSC lines. **Figure S3**. pH and metabolite concentration in media of induced pluripotent stem cells (iPSCs) expanded in two-dimensional planar (2D) and three dimensional suspension (3D) cell culture conditions. **A** pH of cell culture media over time for expanded iPSCs in 2D and 3D conditions (*n* = 3 per group). **C** Glucose concentration of cell culture media over time for expanded iPSCs in 2D and 3D conditions (*n* = 3 per group). **E** Lactate concentration of cell culture media over time for expanded iPSCs in 2D and 3D conditions (*n* = 3 per group). **G** Glutamine concentration of cell culture media over time for expanded iPSCs in 2D and 3D conditions (*n* = 3 per group). **I** Lactate dehydrogenase concentration of cell culture media over time for expanded iPSCs in 2D and 3D conditions (*n* = 3 per group). **K** Ammonia concentration of cell culture media over time for expanded iPSCs in 2D and 3D conditions (*n* = 3 per group). **B** Area under the curve (AUC) for pH, **D** glucose, **F** lactate, **H** glutamine, **J** lactate dehydrogenase and **L** ammonia measurements from day 0 to day 5 from iPSCs cultured in 2D and 3D conditions (*n* = 3 per group). **Figure S4.** Comparison of embryoid bodies generated from iPSCs expanded through 2D planar and 3D suspension culture conditions. **A** Microscopy showing embryoid body morphology and immunohistochemistry of embryoid bodies evaluating ectoderm, mesoderm, and endoderm markers to assess spontaneous differentiation. **B** Transcriptomic assessment of ectoderm, **C** mesoderm, and **D** endoderm gene expression within embryoid bodies generated from 2D planar and 3D suspension conditions and iPSCs expanded using 2D planar and 3D suspension culture conditions. **E** Genetic microarray results comparing the expression of key pluripotency genes among PBMCs and embryoid bodies generated from 2D planar and 3D suspension iPSCs. **F** Differential expression of in 2D and 3D embryoid bodies of primed markers *FGF2*, **G** *DNMT3B* and **H** *IDO1* and **I** naïve markers *GDF3*, **J** *Nanog* and **K** *c-Myc*. **Figure S5.** Flow cytometric cell characterization following 1, 5, and 10 passages using 2D planar and 3D suspension iPSC expansion. Characterization of Oct4, SSEA4, Sox2, Nanog, Tra-1-60, and Tra-1-81 pluripotency markers, CD24, CD130, CD90, CD75, naïve/prime markers, and Ki67 during iPSC expansion using 2D planar and 3D suspension approaches following **A** 1 passage, **B** 5 passages, and **C** 10 passages.  **Figure S6.** Transcript assessment of iPSCs expanded using 2D planar and 3D suspension protocols. Only statistically significant differences are noted within graphs. **Supplementary Tables. Table S1.** Patient demographics used in this study. **Table S2.** Polymerase chain reaction mix used for assessment of viral clearance in iPSCs. *x3.2 reactions were prepared to allow for 1 tube containing the test sample one for the positive control (Beta actin) well and one for the negative control (nuclease free water). **Table S3.** Forward and reverse primer sequences for polymerase chain reaction assessment of viral clearance in induced pluripotent stem cells. These sequences were adapted from CytoTune iPS 2.0 Sendai Reprogramming Kit (Thermo Fisher cat. A16517). **Table S4.** Thermocycler set up for Viral Screening PCR. **Table S5.** Quantitative Polymerase Chain Reaction Sequence for Karyotype Analysis. **Table S6.** Thermo Fisher TaqMan Micro Array configuration. **Table S7.** Sequences and amplicon length of primers used for RT-PCR assessment. **Table S8.** Antibodies and concentrations used for flow cytometry and immunohistochemistry. *All secondaries for flow cytometry were used at a 1:500 concentration and all secondaries for immunohistochemistry were used at a 1:250 concentration.

## Data Availability

The datasets generated and/or analyzed during the current study are available from the corresponding authors upon reasonable request. This paper does not report original code and did not generate new unique reagents.

## Abbreviations

2D: Two-dimensional
3D: Three-dimensional
ALP: Alkaline phosphatase
EDTA: Ethylenediaminetetraacetic acid
gDNA: Genomic DNA
GMP: Good manufacturing practice
h: Hour or hours
H&E: Hematoxylin and eosin
iPSC: Induced pluripotent stem cell
IQR: Interquartile range
PBMC: Peripheral blood mononuclear cells
PBS: Phosphate buffer solution
PFA: Paraformaldehyde
q-PCR: Quantitative PCR
RockI: Rho-kinase inhibitor
rpm: Revolution per minute
rhVTN: Recombinant human Vitronectin
