# Supplementary material for: Suspension culture improves iPSC expansion and pluripotency phenotype
Source: Stem Cell Res Ther. 2023 Jun 6;14:154. doi: 10.1186/s13287-023-03382-9 (PMC10245469; doi:10.1186/s13287-023-03382-9)
Supplement: Supplementary file 1 — Additional file 1. Supplementary Figures. Figure S1. Extended quality control performed on reprogrammed iPSC lines. A Gating strategy used for flow cytometric analysis of the selected iPSC line with isotype control. Briefly, forward and side scatter was used to identify the cell population and remove debris and other events of non-interest based on size and complexity. Width and height of cells was used to exclude the double or multiple cells from single cells. Single cells were selected for further analysis and examined for the expression of Oct4, SSEA4, Nanog and Sox2. Isotype controls were used to accurately gate positive staining and data were acquired using the CytoFLEX S flow cytometer and analysed using the CytExpert software (Beckman Coulter). B Gating strategy for cytometric analysis of Tra-1-60 and Tra-1-81 with single stain results. C Clearance of reprogramming vectors and lack of mycoplasma contamination. To test the absence of the Sendai reprogramming vectors a PCR that detects the Sendai virus genome and the transgenes, was used. PCR products were analysed by 1% agarose gel electrophoresis. iPSCs were tested for the expression of Sev, KOS, KLF4, and c-Myc with β-actin as an internal control. Infected PBMC were used as positive control for transgene presence while un-infected PBMC were used as negative control. Similarly, Mycoplasma PCR Detection Kit was used to detect contamination by 200+ strains of Mycoplasmas. This kit includes a positive Mycoplasma control and water was used as negative control. Full-length blots/gels are presented. Figure S2. Expansion and evaluation of four iPSC lines expanded in 2D planar and 3D suspension cell culture. A Cell size following 3D suspension cluster dissociation and 2D cell passaging on days 0, 3, and 5 of expansion of three iPSC lines. B Absolute cell number expansion using 2D planar and 3D suspension cell culture of iPSC line 1, C iPSC line 2, D iPSC line 3 and E iPSC line 4. F Fold expansion following 3 and 5 da [file 13287_2023_3382_MOESM1_ESM.docx]

**Supplementary Material**

**Supplementary Figures**

**Figure S1. Extended quality control performed on reprogrammed iPSC lines. A** Gating strategy used for flow cytometric analysis of the selected iPSC line with isotype control. Briefly, forward and side scatter was used to identify the cell population and remove debris and other events of non-interest based on size and complexity. Width and height of cells was used to exclude the double or multiple cells from single cells. Single cells were selected for further analysis and examined for the expression of Oct4, SSEA4, Nanog and Sox2. Isotype controls were used to accurately gate positive staining and data were acquired using the CytoFLEX S flow cytometer and analysed using the CytExpert software (Beckman Coulter). **B** Gating strategy for cytometric analysis of Tra-1-60 and Tra-1-81 with single stain results. **C** Clearance of reprogramming vectors and lack of mycoplasma contamination. To test the absence of the Sendai reprogramming vectors a PCR that detects the Sendai virus genome and the transgenes, was used. PCR products were analysed by 1% agarose gel electrophoresis. iPSCs were tested for the expression of Sev, KOS, KLF4, and c-Myc with β-actin as an internal control. Infected PBMC were used as positive control for transgene presence while un-infected PBMC were used as negative control. Similarly, Mycoplasma PCR Detection Kit was used to detect contamination by 200+ strains of Mycoplasmas. This kit includes a positive Mycoplasma control and water was used as negative control. Full-length blots/gels are presented.

**Figure S2. Expansion and evaluation of four iPSC lines expanded in 2D planar and 3D suspension cell culture.** A) Cell size following 3D suspension cluster dissociation and 2D cell passaging on days 0, 3, and 5 of expansion of three iPSC lines. B) Absolute cell number expansion using 2D planar and 3D suspension cell culture of iPSC line 1, C) iPSC line 2, D) iPSC line 3 and E) iPSC line 4. F Fold expansion following 3 and 5 days of cell expansion in 2D planar and 3D suspension cell culture of four iPSC lines.

**Figure S3. pH and metabolite concentration in media of induced pluripotent stem cells (iPSCs) expanded in two-dimensional planar (2D) and three-dimensional suspension (3D) cell culture conditions.** A) pH of cell culture media over time for expanded iPSCs in 2D and 3D conditions (n = 3 per group). C) Glucose concentration of cell culture media over time for expanded iPSCs in 2D and 3D conditions (n = 3 per group). E) Lactate concentration of cell culture media over time for expanded iPSCs in 2D and 3D conditions (n = 3 per group). G) Glutamine concentration of cell culture media over time for expanded iPSCs in 2D and 3D conditions (n = 3 per group). I) Lactate dehydrogenase concentration of cell culture media over time for expanded iPSCs in 2D and 3D conditions (n = 3 per group). K) Ammonia concentration of cell culture media over time for expanded iPSCs in 2D and 3D conditions (n = 3 per group). B) Area under the curve (AUC) for pH, D) glucose, F) lactate, H) glutamine, J) lactate dehydrogenase and L) ammonia measurements from day 0 to day 5 from iPSCs cultured in 2D and 3D conditions (n = 3 per group).

**Figure S4.** **Comparison of embryoid bodies generated from iPSCs expanded through 2D planar and 3D suspension culture conditions**. A) Microscopy showing embryoid body morphology and immunohistochemistry of embryoid bodies evaluating ectoderm, mesoderm, and endoderm markers to assess spontaneous differentiation. B) Transcriptomic assessment of ectoderm, C) mesoderm, and D) endoderm gene expression within embryoid bodies generated from 2D planar and 3D suspension conditions and iPSCs expanded using 2D planar and 3D suspension culture conditions. E) Genetic microarray results comparing the expression of key pluripotency genes among PBMCs and embryoid bodies generated from 2D planar and 3D suspension iPSCs. F) Differential expression of in 2D and 3D embryoid bodies of primed markers *FGF2*, G) *DNMT3B* and H) *IDO1* and I) naïve markers *GDF3*, J) *Nanog* and K) *c-Myc*.

**Figure S5.** **Flow cytometric cell characterization following 1, 5, and 10 passages using 2D planar and 3D suspension iPSC expansion.** Characterization of Oct4, SSEA4, Sox2, Nanog, Tra-1-60, and Tra-1-81 pluripotency markers, CD24, CD130, CD90, CD75, naïve/prime markers, and Ki67 during iPSC expansion using 2D planar and 3D suspension approaches following A) 1 passage, B) 5 passages, and C) 10 passages.

**Figure S6. Transcript assessment of iPSCs expanded using 2D planar and 3D suspension protocols.** Only statistically significant differences are noted within graphs.

**Supplementary Tables**

**Table S1. Patient demographics used in this study.**

| **iPSC line** | **Age** | **Sex** | **Gender** | **Health status** |
| --- | --- | --- | --- | --- |
| **#1** | 53 | Female | Female | Healthy |
| **#2** | 43 | Female | Female | Healthy |
| **#3** | 28 | Male | Male | Healthy |
| **#4** | 29 | Male | Male | Healthy |

**Table S2. Polymerase chain reaction mix used for assessment of viral clearance in iPSCs.**
*x3.2 reactions were prepared to allow for 1 tube containing the test sample one for the positive control (Beta actin) well and one for the negative control (nuclease free water).

| Reagent | 10 µL total volume | x3.2 reactions |
| --- | --- | --- |
| *Template DNA | 1.0 µL | - |
| Primers F & R | 2.0 µL | 6.4 µL |
| Master Mix | 5.0 µL | 16.0 µL |
| Nuclease free water | 2.0 µL | 6.4 µL |
| Total Volume: | 10 uL | 32 uL |

**Table S3. Forward and reverse primer sequences for polymerase chain reaction assessment of viral clearance in induced pluripotent stem cells.** These sequences were adapted from CytoTune iPS 2.0 Sendai Reprogramming Kit (Thermo Fisher cat. A16517).

| Gene ID | Forward Primer | Reverse Primer | Product Size (base pair) |
| --- | --- | --- | --- |
| *SEV* | GGA TCA CTA GGT GAT ATC GAG C | ACC AGA CAA GAG TTT AAG AGA TAT GTA TC | 181 |
| *KOS* | ATG CAC CGC TAC GAC GTG AGC GC | ACC TTG ACA ATC CTG ATG TGG | 528 |
| *CMYC* | TAA CTG ACT AGC AGG CTT GTC G | TCC ACA TAC AGT CCT GGA TGA TGA TG | 532 |
| *KLF* | TTC CTG CAT GCC AGA GGA GCC C | AAT GTA TCG AAG GTG CTC AA | 410 |
| *Human β-Actin* | TGC CCA TTT ATG AGG GCT AC | GCC ATC TCG TTC TCG AAG TC | 195 |

**Table S4. Thermocycler set up for Viral Screening PCR.**

| Temperature | Duration | Cycles |
| --- | --- | --- |
| 95° | 5 minutes | 1 |
| 95° | 30 seconds | 34 |
| 55° | 30 seconds |  |
| 72° | 30 seconds |  |
| 72° | 5 minutes | 1 |

**Table S5. Quantitative Polymerase Chain Reaction Sequence for Karyotype Analysis.**

| Stage | Cycles | Temperature (°C) | Cycling Time (min:sec) |
| --- | --- | --- | --- |
| Polymerase Activation | 1 | 95.0 | 3:00 |
| Denature | 40 | 95.0 | 0:05 |
| Anneal |  | 60.0 | 0:30 |

**Table S6. Thermo Fisher TaqMan Micro Array configuration.**

| #Thermo Fisher Array Export# | |  |  |  |  |  |
| --- | --- | --- | --- | --- | --- | --- |
| TaqMan Array Micro Fluidic Cards:48 | |  |  |  |  |  |
| Assay ID | **Gene** | **Gene Name(s)** | **Species** | **Amplicon Length** | **Best Coverage** | **3' Most** |
| Hs01053790_m1 | ABCG2 | ATP binding cassette subfamily G member 2 (Junior blood group) | Human | 83 | Yes | No |
| Hs00923299_m1 | ACVR1B | activin A receptor type 1B | Human | 74 | Yes | No |
| Hs00609603_m1 | ACVR2B | activin A receptor type 2B | Human | 101 | Yes | No |
| Hs01029144_m1 | ALPL | alkaline phosphatase |  |  |  |  |
| Hs00187842_m1 | B2M | beta-2-microglobulin | Human | 64 | Yes | No |
| Hs00204257_m1 | CD274 | CD274 molecule | Human | 77 | Yes | Yes |
| Hs01023895_m1 | CDH1 | cadherin 1 | Human | 80 | Yes | No |
| Hs00172106_m1 | CIITA | class II |  |  |  |  |
| Hs00175480_m1 | CTLA4 | cytotoxic T-lymphocyte associated protein 4 | Human | 93 | Yes | No |
| Hs00607978_s1 | CXCR4 | C-X-C motif chemokine receptor 4 | Human | 153 | Yes | Yes |
| Hs99999905_m1 | GAPDH | - | Human | 0 | No | No |
| Hs00999691_m1 | FGF4 | fibroblast growth factor 4 | Human | 130 | No | No |
| Hs00915142_m1 | FGFR1 | fibroblast growth factor receptor 1 | Human | 62 | No | No |
| Hs00232764_m1 | FOXA2 | forkhead box A2 | Human | 66 | No | No |
| Hs00231106_m1 | FOXO1 | forkhead box O1 | Human | 103 | Yes | Yes |
| Hs01106466_s1 | FUT4 | fucosyltransferase 4 | Human | 152 | Yes | Yes |
| Hs00171403_m1 | GATA4 | GATA binding protein 4 | Human | 68 | Yes | No |
| Hs00220998_m1 | GDF3 | growth differentiation factor 3 | Human | 65 | Yes | Yes |
| Hs01058806_g1 | HLA-A | major histocompatibility complex |  |  |  |  |
| Hs00818803_g1 | HLA-B | major histocompatibility complex |  |  |  |  |
| Hs00167041_m1 | HNF1A | HNF1 homeobox A | Human | 96 | Yes | No |
| Hs00230853_m1 | HNF4A | hepatocyte nuclear factor 4 alpha | Human | 49 | Yes | No |
| Hs99999909_m1 | HPRT1 | hypoxanthine phosphoribosyltransferase 1 | Human | 100 | No | Yes |
| Hs00961622_m1 | IL10 | interleukin 10 | Human | 74 | Yes | Yes |
| Hs00174131_m1 | IL6 | interleukin 6 | Human | 95 | Yes | Yes |
| Hs00235006_m1 | ITGA1 | integrin subunit alpha 1 | Human | 87 | Yes | No |
| Hs01041011_m1 | ITGA6 | integrin subunit alpha 6 | Human | 64 | Yes | No |
| Hs00174029_m1 | KIT | KIT proto-oncogene receptor tyrosine kinase | Human | 64 | Yes | No |
| Hs00358836_m1 | KLF4 | Kruppel like factor 4 | Human | 110 | Yes | Yes |
| Hs00702808_s1 | LIN28A | lin-28 homolog A | Human | 143 | Yes | Yes |
| Hs00153408_m1 | MYC | v-myc avian myelocytomatosis viral oncogene homolog | Human | 107 | Yes | Yes |
| Hs04260366_g1 | NANOG | Nanog homeobox | Human | 99 | No | Yes |
| Hs00240871_m1 | PAX6 | paired box 6 | Human | 76 | No | No |
| Hs00236830_m1 | PDX1 | pancreatic and duodenal homeobox 1 | Human | 73 | Yes | Yes |
| Hs01574644_m1 | PODXL | podocalyxin like | Human | 82 | Yes | Yes |
| Hs00210532_m1 | PODXL2 | podocalyxin like 2 | Human | 73 | Yes | No |
| Hs04260367_gH | POU5F1 | POU class 5 homeobox 1 | Human | 77 | Yes | Yes |
| Hs00751752_s1 | SOX17 | SRY-box 17 | Human | 149 | Yes | Yes |
| Hs01053049_s1 | SOX2 | SRY-box 2 | Human | 91 | Yes | No |
| Hs00165814_m1 | SOX9 | SRY-box 9 | Human | 102 | Yes | Yes |
| Hs00266645_m1 | FGF2 | fibroblast growth factor 2 | Human | 82 | Yes | No |
| Hs00911929_m1 | TBX2 | T-box 2 | Human | 60 | Yes | No |
| Hs00972656_m1 | TERT | telomerase reverse transcriptase | Human | 79 | No | No |
| Hs00907219_m1 | TPBG | trophoblast glycoprotein | Human | 100 | No | No |
| Hs00864535_s1 | UTF1 | undifferentiated embryonic cell transcription factor 1 | Human | 102 | Yes | Yes |
| Hs01938187_s1 | ZFP42 | ZFP42 zinc finger protein | Human | 146 | Yes | Yes |
| Mr04269880_mr | SEV | Sendai | Markers & Reporters | 59 | No | No |
| Mr04421257_mr | SEV-KOS | Sendai-KLF4-KOS | Markers & Reporters | 80 | No | No |

**Table S7. Sequences and amplicon length of primers used for RT-PCR assessment.**

| **Gene** | **Forward Primer** | **Reverse Primer** | **Amplicon Length** |
| --- | --- | --- | --- |
| XIST | GTTAGGGACAGTGAGTTAGAAATTGT | CTGGACTCAGTAACACCCCTTTC | 512 |
| DNMT3B | CTGGCGTCTGAGCCTTCG | ATTGAGATGCCTGGTGTCTCC | 268 |
| CD31 | CTGAGGAATTGCTGTGTTCTGTG | CTGCTTTGCATTTTCTTTGAGAAGTG | 274 |
| TBXT | CCAGTGCGTTCAGCATCG | CTACCAAGAGCTGCCTCCAC | 254 |
| Pax6 | CACTTAAAAGTGATGGGATTGACTGTCT | ACAGCCCTCACAAACACCTAC | 244 |
| NES | CACCCCTAAGTCCCCAGTG | GGAGCAGTCTGAGGAAGTGG | 234 |
| OTX2 | CCCTCTAAGGCCCTTCGTTTT | GCTTGGATTATAAGGACCAAACTGC | 266 |
| IDO1 | CCCTGTGATAAACTGTGGTCACT | CCACAGTTGTTCAGTAGAAGTTAACTTG | 274 |

**Table S8.** **Antibodies and concentrations used for flow cytometry and immunohistochemistry.***All secondaries for flow cytometry were used at a 1:500 concentration and all secondaries for immunohistochemistry were used at a 1:250 concentration.

| Antibody | Fluorophore | Primary Antibody Supplier (catalog number) | Secondary Antibody Supplier (catalog number) | Dilution for flow cytometry | Dilution for immunohistochemistry |
| --- | --- | --- | --- | --- | --- |
| Tra-1-60 | FITC | Invitrogen (A25617) | - | 1:100 | 1:100 |
| Tra-1-81 | Cy3 | EMD Millipore (MAB4381C3) | - | 1:100 | 1:100 |
| Oct4 | BV421 | EMD Millipore (MAB4419A4) | - | 1:100 | 1:100 |
| Nanog | PE | Invitrogen (PA5-46891) | - | 1:100 | N/A |
| Nanog | FITC | EMD Millipore (MABBD24A4) | - | N/A | 1:100 |
| Sox2 | FITC | Invitrogen (53-9811-82) | - | 1:100 | 1:100 |
| Ki-67 | PerCP-Cy 5.5 | BD (561284) | - | 1:50 | N/A |
| Ki-67 | Secondary PE | Abcam (ab15580) | Sigma (A11036) | N/A | :50 |
| SSEA4 | Secondary APC | Invitrogen (MA1-021) | Jackson Immuno (115-135-164) | 1:100 | 1:100 |
| CD-24 | BV-786 | BD (740971) | - | 1:100 | 1:100 |
| CD130 | BB700 | BD (746079) | - | 1:100 | 1:100 |
| PAX6 | Secondary: APC | Fisher (42-6600) | (Jackson Immuno) 115-135-164 | N/A | 1:200 |
| CD184 | BV421 | BD (562448) | - | N/A | 1:100 |
| CD31 | Secondary PE | Abcam (ab28364) | Sigma (A11036) | N/A | 1:50 |
| Sox17 | Secondary FITC | R&D (963121) | Thermo (A16000) | N/A | 1:10 |
| FoxA2 | Secondary PE | Abcam (108422) | Sigma (A11036) | N/A | 1:50 |
| Otx2 | Secondary FITC | R&D (963273) | Thermo (A16000) | N/A | 1:10 |
| TBXT | Secondary FITC | R&D (963427) | Thermo (A16000) | N/A | 1:10 |
